# Persistent Organic Pollutants and Suspended Particulate Matter in Snow of Eastern Siberia in 2009–2023: Temporal Trends and Effects of Meteorological Factors and Recultivation Activities at Former Industrial Area

**DOI:** 10.3390/toxics12010011

**Published:** 2023-12-22

**Authors:** Elena A. Mamontova, Alexander A. Mamontov

**Affiliations:** Vinogradov Institute of Geochemistry SB RAS, Irkutsk 664033, Russia; mamontov@igc.irk.ru

**Keywords:** snow, particulate matter, PCB, OCP, Eastern Siberia, temporal trends, meteorological factors, former organochlorine industrial area

## Abstract

Suspended particulate matter (SPM), polychlorinated biphenyls (PCBs), and organochlorine pesticides (OCP) were studied in the snow cover at urban and suburban localities in the Irkutsk region, Eastern Siberia for their temporal variations in 2009–2023, daily deposition fluxes (DDFs), and effects of some meteorological factors, as well as the effects of different technogenic activities in the industrial area of the former organochlorine enterprises of Usol’ekhimprom. SPM loads at both stations were found to be at a low level of pollution. The levels of HCB, α + γ-HCH, and ∑*p*,*p*′-DDX were lower than Russian maximum permissible levels (MPLs) in drinking water, groundwater, and surface water for household drinking and cultural purposes. The sums of all organochlorine compounds studied in snow were higher than the MPL in freshwater water bodies for fishery purposes. The levels of the DDFs of HCHs, DDTs, and heptachlorinated PCB decreased, di- and trichlorinated PCB levels increased, and HCB levels changed at a polynomial line during 2009–2023. The change in the relative composition of PCBs was found as a result of recultivation activities at the industrial area of the former organochlorine enterprise of Usol’ekhimprom. The air humidity and temperature are the key meteorological factors affecting the DDFs of PCBs and OCPs.

## 1. Introduction

Snow cover is an important climate-forming factor in regions where snow cover lasts for several months [1]. Snow cover is one of the major sources of soil moisture, especially in areas with low precipitation volumes. In winter, the snow cover protects the soil from sharp temperature fluctuations due to its low thermal conductivity [1].

The atmospheric scavenging of contaminants and their temporal deposition in the snow cover is another important function of snow [2,3,4,5]. Falling snowflakes effectively scavenge atmospheric particulate matter and organic and inorganic compounds in the gas phase of the air and those adsorbed on the particles [2,3,4,5,6,7,8,9]. The snow cover can be used as an indicator of air pollution [5,7,10]. Later, these contaminants accumulate in the snow cover during a cold season until spring in temperate latitudes or during a long period in the preserved non-melting snow cover in the Arctic and Antarctic areas [6,11,12,13,14]. In spring, snowmelt water brings pollutants to the soil and water bodies by facilitating the distribution and introduction of pollutants into terrestrial and aquatic food webs [15]. This leads to an increase in the body burden of pollutants on living organisms and, possibly, to various negative health effects in terrestrial and waterbody biota and humans [16,17].

Thus, snow cover formed during the cold season can serve as an indirect indicator of atmospheric pollution through the presence of emerging and legacy contaminants including both organic and inorganic compounds as well as suspended particulate matter. Polychlorinated biphenyls (PCBs) and organochlorine pesticides (OCPs) are among these legacy contaminants.

PCBs and OCPs are included in the group of persistent organic pollutants (POPs) that are widely distributed in environmental media and affect the health of humans and other living organisms [17,18]. POPs enter the environment from industrial sources, either during their target production or as by-products, or as a result of agricultural applications [18,19,20]. The secondary sources of POP emissions are contaminated technogenic soils, buildings constructed in industrial areas after the shutting down of enterprises, and the untreated industrial waste located on land. Earlier studies [21,22] focused on the effects of emissions from the chemical enterprise of Usol’ekhimprom, which is the industrial source of POPs including PCBs, hexachlorobenzene (HCB), and polychlorinated dibenzo-p-dioxins and dibenzofurans (PCDD/Fs) in the Usol’e-Sibirskoe area, on the terrestrial environment of the town of Usol’e-Sibirskoe and adjacent territories.

The aim of the present study was to estimate the temporal variations in the contents of PCBs and OCPs in the snow cover at two long-term observation stations of organochlorines in environmental media of Eastern Siberia during 2009–2023 period taking into account changes in meteorological factors and industrial activities.

## 2. Materials and Methods

### 2.1. Study Site

Snow sampling stations are located in the River Angara valley in the southern part of Irkutsk Region, Eastern Siberia. The climate here is sharply continental. Winter is severe, with weak winds and frequent inversions of air temperature [23,24]. In the town of Irkutsk, the coldest month of the year is January (mean temperature is –20.6 °C). The predominant wind directions are western and northwestern [25,26]. In winter, the anticyclone results in small precipitation volume. Meteorological conditions vary considerably from year to year, which affect the dates of formation and destruction of snow cover. The deviation of the average data on formation and destruction of stable snow cover is 15–30 days [27]. The first unsteady snow cover appears in the first or second decades of October. A stable snow cover forms about one month later. The greatest thickness of snow cover is observed in late February–early March. During this time, insolation increases and western cyclones bring warmth to the region, which leads to the destruction of stable snow cover in the second half of March [27].

The southern part of Irkutsk Region is the most populated industrial area, uniting the towns of Irkutsk, Schelekhov, Angarsk, and Usol’e-Sibirskoe. The total population of these four towns comprises 1,108,400 people, including 617,000 people living in the town of Irkutsk, which is the center of Irkutsk Region [28,29]. The population of these towns and surrounding settlements is experiencing increased burden on health due to exposure to emerging contaminants contained in emissions from transport and stationary industrial sources [30,31]. The area is the zone of intense industrial activities including chemical industry, oil refining, non-ferrous metallurgy, machine building, and the production of building materials, as well as heat and power plants [28]. Some of these enterprises are potential sources of POPs. Chemical industry at Usol’ekhimprom, located on the windward side of all settlements of this industrial area, brings pollution with PCBs, HCB, and PCDD/Fs over a considerable area [22,23,32].

Ethyl fluid was the first product of Usol’ekhimprom in 1936. The organochlorine production at the former enterprises of Usol’ekhimprom started in 1943 with production of caustic soda and ethyl chloride [33]. The Usol’ekhimprom enterprise produced more than hundred named chemical substances (chloronaphthalene, HCl, vinyl chloride, household chemicals, methylcellulose trichlorethylene, penatachlorobenzene, epichlorohydrin, epoxydianic resin, varnishes, enamels, etc.) [33]. Twelve thousand people worked at the enterprise. The total area of the enterprises was 610 hectares [34]. In 1998, the mercury electrolysis facility was shut down due to the high pollution of the surrounding area with mercury [35]. In 2012, the industrial area of Usol’ekhimprom was mothballed and the enterprise was declared bankrupt [34]. Due to the threat of chemical pollution, a municipal and then regional state of emergency were introduced in Usol’e-Sibirskoe town in 2018 and 2020, respectively [34]. In the fall of 2020, efforts to eliminate the accumulated environmental damage in the industrial area of Usol’ekhimprom started [34,36]. In 2020, the above-ground part of the mercury electrolysis workshop building was dismantled, containers with some toxic wastes were brought to a safe state, and two wells for deep burial of epichlorohydrin waste were pumped out and preserved [34]. In 2021–2023, other workshop buildings were dismantled. In 2023, work on the reclamation of the sludge collector began [34].

### 2.2. Snow Sampling

**Figure 1:**
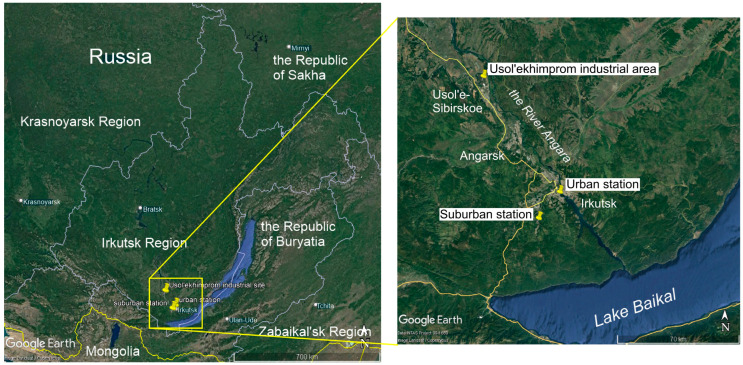
The scheme of study area and the location of snow sampling stations.

Snow samples were collected every winter in the 2009–2023 period before the beginning of intensive snowmelt, in the period from 16 February to 3 March, at the urban (52°14′ N, 104°16′ E) and suburban (52°6′ N, 104°5′ E) stations (Figure 1, Table S1). The urban station lies in the recreational zone of the town of Irkutsk. The suburban station is located 20 km from the urban station in a locality used for summer vacationing and recreation and/or production of organic food as a hobby and for the town’s own consumption. The sampling was performed either under snow sampling companies in the southern part of Irkutsk Region in 2009–2011, 2016, and 2021 [37,38] or as a study of the PCB and OCP temporal trends at the only target sampling sites during the remaining years (Table S1).

The snow samples were collected using a metal shovel to the depths of the snow cover in a polyethylene bag. The height of the snow cover and the square of sampling core, as well as the weight of the snow samples, were measured on the day of sampling and were used to calculate the snow density and snow water equivalent (SWE).

### 2.3. Analysis of PCBs and OCPs

#### 2.3.1. Sample Pretreatment and Instrumental Analysis of PCBs and OCPs

OCP and PCB analyses were carried out in the laboratory of the Institute of Geochemistry in Irkutsk, Russia.

Snow samples were melted at room temperature on the day of the sampling. For melting, a snow sample was placed in a metal reservoir, which had previously been washed with soap and water and rinsed with *n*-hexane:acetone (1:1). An amount of 0.9 L of snow water was placed into a dark glass bottle. Surrogate (PCB-14) and internal (PCB-65) standards were added in snow water. Then, snow water was extracted thrice using 20 mL, 15 mL and 15 mL dichloromethane. Extracts of the snow samples were dried over Na_2_SO_4_. After the extraction, the solvent was evaporated. The snow sample analyses in 2009–2017 and 2018–2023 were accomplished using the methods described in [39] and [38], respectively. Briefly, in 2009–2017, the extracts of snow samples were cleaned using one liquid chromatography column containing silica gel, aluminum oxide, and Na_2_SO_4_. In 2018–2023, gel permeation chromatography column filled with bio-bead S-X3 was added during the clean-up procedure to eliminate interferences shown in former samples. The fraction containing PCBs and OCPs was evaporated to 30 μL using a stream of nitrogen.

In 2009–2017 and 2018–2023, OCP and PCB analyses were performed using GC/ECD and GC/MC, respectively using the method described in detail in [38,39].

The GC/ECD analyses were performed on an HP 5890A Series II gas chromatograph (Agilent Technologies, Santa Clara, CA, USA) equipped with DB-5 capillary columns (J&W Scientific, 0.25 μm film thickness, 0.25 mm inner diameter, 60 m length).

GC/MC analyses were performed on a Chromatec Crystal 5000 (UniChrom, Lynnwood, WA, USA) gas chromatograph with a mass spectrometer operating in a selected ion monitoring (SIM) and electronic ionization mode equipped with a DB-5 capillary column (J&W Scientific; a 0.25 μm film thickness, a 0.25 mm inner diameter, and a 60 m length) in 2018–2021 or Rxi-5 ms capillary column (Restek; a 0.25 μm film thickness, a 0.25 mm inner diameter, and a 60 m length) in 2022–2023. The results of GC/ECD analyses of indicator PCBs, *p*,*p*′-DDTs, and HCHs, were confirmed in snow sampled in 2018–2019 using improved clean-up procedure and a GC/MS analyses (Chromatec Crystal 5000 gas-chromatograph-equipped mass-spectrometer).

All the samples were analyzed for up to 37 individual and coeluting congeners of PCBs including indicator PCB congeners (IUPAC no.: 28, 52, 101/90, 153, 138, and 180), HCB, *p*,*p*′-dichlorodiphenyltrichloroethane (*p*,*p*′-DDT), *p*,*p*′-dichlorodiphenyldichloroethane (*p*,*p*′-DDD), *p*,*p*′-dichlordiphenyldichlorethylene (*p*,*p*′-DDE), and α- and γ-isomers of hexachlorocyclohexane (HCH) in 2009–2017 (Table S1). In 2018–2023, *o*,*p*′-dichlorodiphenyltrichloroethane (*o*,*p*′-DDT), *o*,*p*′-dichlorodiphenyldichloroethane (*o*,*p*′-DDD), and *o*,*p*′-dichlordiphenyldichlorethylene (*o*,*p*′-DDE) were added to the list of compounds analyzed (Table S1).

Standards of individual PCB congeners and the PCB and the OCP mixtures were obtained from the Dr. Ehrenstorfer Laboratory (Ausburg, Germany). Aluminum oxide and silica gel for column chromatography were purchased from MERCK (Darmstadt, Germany). All solvents used were purified and controlled for interference prior to use.

#### 2.3.2. Quality Control and Quality Assurance (QA/QC)

Method recoveries were defined using spiked samples. They lay between 80% and 120% for most compounds, with the only exception being that of PCB-28 (137%) for the method described in [39], and between 70% and 130% for OCPs and 77% and 117% for PCBs, with the only exceptions being those of *p*,*p*′-DDD (161%), *o*,*p*′-DDE (178%), *o*,*p*′-DDD (142%), and PCB-47 (67%) for the method described in [38]. Corrections for recoveries were not made.

Laboratory blanks to estimate the purity of reagents and instruments were run with each series of 10–12 samples in the big snow study/under a sampling company. In other years, when snow was sampled from only two stations, two laboratory blanks were run with two samples. Only samples in which the level of the analyzed compound exceeded the level of the blank by 3.5 times were taken into consideration. Corrections for blanks were not performed.

Method detection limits (MDLs) were received from the blanks and quantified as the averages of blanks plus three standard deviations. In 2009–2017, MDLs varied between 0.001 and 1.4 ng/L for OCPs and between 0.001 and 1.3 ng/L for PCBs. In 2018–2023, MDLs varied between 0.003 and 0.069 ng/L for OCPs and between 0.001 and 2.3 ng/L for PCBs (depending on the congener). Concentrations below MDL were replaced by ½ MDL for the calculation of the sum, average, median, and standard deviation and for statistical analysis.

Recoveries for the PCB-14 surrogate equaled 56–114% (mean: 88%) in 2009–2017 and 53–127% (mean: 94%) in 2018–2023. No correction was made to the samples.

### 2.4. Analysis of Suspended Particulate Matter

The suspended particulate matter (SPM) in snow water was analyzed using the gravimetric method described in [38,40,41], which involves filtration through nitrate cellulose membrane filters with a pore diameter of 0.45 μm and filter diameter of 35 mm (VLADiSART, FMNC-0.45 (11306)).

### 2.5. Data Analysis

SPM and POP values were discussed in terms of concentrations of mg or ng per liter and deposition rate (DR) of mg or ng of substances per square meter (m^2^) obtained from concentrations of compounds per liter using Equation (1):

(1) $$DR=\frac{C\times V}{S},$$

Here, *C* is concentration of compounds (ng(POPs)/L or mg(SPM)/L), *V* is whole volume of snow sample (L), and *S* is the square of snow sampling (m^2^).

The daily deposition fluxes for POPs (2) and suspended particulate matter load (3) were calculated by dividing the load/deposition rate by the number of days of stable snow cover before snow sampling [42]:

(2) $$DDF=\frac{{DR}_{POPs}}{n},$$

(3) $${Load}_{SPM}=\frac{{DR}_{SPM}}{n}$$

where DDF is daily deposition flux of POPs (ng/m^2^ per day), Load_SPM_ is daily SMP load (or dust load) (mg/m^2^ per day), DR_POPs_ is POP deposition rate (ng/m^2^), DR_SPM_ is SPM deposition rate (mg/m^2^), and *n* is the number of days from the date of a stable snow cover formation to the date of snow sampling.

### 2.6. Statistical Analysis

Statistical analyses were accomplished using STATISTICA 6.0 software for Windows (StatSoft, Inc., Tulsa, OK, USA). The concentrations of PCBs, OCPs, and particulate matter were presented as means and medians, with the standard deviations (SD), standard errors (SE), and ranges. The data were ln-transformed before statistical analyses. The significances of the differences in the DDFs of POPs and SPM load at the urban and suburban stations and between those during different temporal periods were assessed using Student’s *t*-test in independent groups. The Pearson test was carried out to evaluate the correlations between compounds found in the samples of snow. Temporal trends were investigated using a linear regression analysis comparing ln-transformed SPM load and DDFs of POPs and the years of investigation. The relative PCB homological patterns were studied using the cluster method.

Linear and multiple regressions were used to analyze the effects of the complex of meteorological factors on daily deposition fluxes of POPs and SPM load in winter. The complex of meteorological factors included average temperature, precipitation volume, air humidity, and sums of sunshine durations for the period of the stable snow cover before snow sampling (November (Nov) + December (Dec) of previous year before sampling + January (Jan) + February (Feb) of the year of sampling), as well as for every month separately (Nov, Dec, Jan, and Feb). Meteorological parameters including the monthly temperature, precipitation volume, air humidity, and sunshine duration, and the characteristics of the snow cover (the date of a stable snow cover formation) at the meteorological station in the town of Irkutsk were obtained from the Federal Service for Hydrometeorology and Environmental Monitoring website (Roshydromet) [43,44,45,46,47].

The effects of the total volume of annual emissions into atmosphere, from stationary sources in Irkutsk region and separately in the nearest towns of Irkutsk, Angarsk, Usol’e-Sibirskoe, and Schelekhov in year preceding the snow sampling events, together with SPM load on the DDFs of POPs, were also studied using regression analyses. The volumes of annual emissions into atmosphere from stationary sources in Irkutsk region and in the towns of Irkutsk, Angarsk, Usol’e-Sibirskoe, and Schelekhov were downloaded from Federal State Statistics Service [29,48,49,50,51,52,53,54,55,56,57,58].

A confidence level of *p* < 0.05 was applied as the criterion for statistical significance.

### 2.7. Backward and Forecast Trajectories Analysis

In order to assess the origin of air masses arriving at the urban and suburban sampling sites, 48 h forecast trajectory emissions from land source in Usol’ekhimprom industrial area and backward trajectories for urban and suburban sampling sites for days with the highest volumes of snow precipitation in winter in 2020–2021 [59] were studied using the Hybrid Single-Particle Lagrangian Integrated Trajectory (HYSPLIT data available at http://ready.arl.noaa.gov/HYSPLIT.php (accessed on 28 July 2023) modeling system created by the National Oceanic and Atmospheric Administration’s Air Resources Laboratory [60,61].

## 3. Results and Discussion

### 3.1. Backward and Forecast Air Trajectories

It was found that according to the performed forecast (Figure S1) and the backward (Figures S2 and S3) air trajectory models, the northern, northwestern, and western directions were the predominant directions of air masses during winter at the urban and suburban stations, confirming the conclusions made by [25] and suggesting the effect of the emissions from the Usol’ekhimprom industrial area on the southern part of the Irkutsk Region including the stations under study. More information on the results of backward and forecast air trajectory modeling is presented in the Supplementary Materials (Text S1).

### 3.2. Characteristics of Some Meteorological Indexes in 2009–2023 and Snow Cover at the Time of Sampling

The characteristics of snow cover, including height, density, and snow water equivalent (SWE), are presented in Table 1.

The average monthly temperature and duration of sunshine in Irkutsk in the period of the snow study in January 2009–February 2023 [43,46] were comparable to the Climatological Standard Normals for 1991–2020 [62] and slightly higher than the Climatological Reference Normals for 1961–1990 [63] at this station (Figure S4). The average monthly precipitation volumes in 2009–2023 [44] were comparable to both Climatological Standard and Reference Normals [62,63].

The means of the snow cover height and snow water equivalent (SWE) for the area investigated (Table 1) were typical of the town of Irkutsk and the Irkutsk area [23,24]. The height and SWE were usually slightly higher at the suburban station than at the urban station. And, on the contrary, the density of snow at the urban station was slightly higher than at the suburban station. The same variations in the height and density of snow cover were found in Tyumen and the surrounding area [64] as a result of snow melting in the urban area due to the “urban heat island” effect [65].

There were interannual variations in meteorological indexes, especially in the winter months during the study period of January 2009–February 2023 (Figure S5), that resulted in considerable interannual fluctuations of snow cover characteristics (Figure S6).

More information on the characteristics of snow cover and meteorological indexes can be found in the Supplementary Materials (Text S2).

### 3.3. Suspended Particulate Matter in Snow

The concentrations of suspended particulate matter (SPM) varied from 24 mg/L to 137 mg/L and from 1.8 to 38 mg/L at the urban and suburban stations, respectively (Table 1). The mean level of SPM in snow at the urban station for the study period amounted to 66 mg/L, which corresponded to the SPM mean for the southern area of the Irkutsk Region that was found in 2021 (61 mg/L [38]), and it was five times higher than that at the suburban station (12.8 mg/L [38]). The mean SPM level in snow at the urban station in the town of Irkutsk was lower than that in snow at the city of Khabarovsk (Russian Far East), 122 mg/L [66], and higher than the levels found in other Russian towns—Barnaul (Altay Krai, Western Siberia), 10.8 mg/L [67]; Tyumen (Tyumen Region, Western Siberia), 37.1 mg/L [64]; Izhevsk (the Udmurt Republic, Ural area), 26.4 mg/L [68]; and Nizhnevartovsk (the Khanty-Mansi Autonomous Okrug, Western Siberia), 17.2 mg/L [69]—and in natural reserves in the Russian Far East, where the values were 16 and 31 mg/L [66]. The mean SPM concentrations at both stations investigated were higher than those found in snow from high mountainous areas in Europe (up to 3.5 mg/L [70,71]); remote areas in the Ob River catchment (Western Siberia), where it was 1.47 mg/L [67]; and rural and background areas in the Khanty-Mansi Autonomous Okrug (8.4 mg/L [72] and Tyumen Region (7.5 mg/L [64]).

The deposition rate (DR) of SPM varied from 115 to 1273 mg/m^2^ and from 1176 to 6900 mg/m^2^ at the suburban and urban stations, respectively. The mean DR of SPM at the suburban station (653 mg/m^2^) was higher than the DR of SPM in the background area, and it was lower than that in the urban area in Western Siberia (74 and 1492 mg/m^2^, respectively [69]). The mean DR of SPM at the urban station (3196 mg/m^2^) was higher as compared to both these values.

Taking into account the differences in the dates of the formation and destruction of stable snow cover between regions of the world, as well as the interannual fluctuations of these dates, the daily SPM load (mg/m^2^ per day) is more acceptable for comparison between regions and for the evaluation of temporal trends. The SPM load is also used as an indirect indicator in the assessment of air pollution at settlements [42,73].

The SPM load at the suburban station (6.5 (1.26–11.6) mg/m^2^ per day) was comparable with that in background and rural areas in the Tyumen Region, where it was 5.5 (2.4–8.3) mg/m^2^ per day [64]; Omsk Region, where it was 3.1 mg/m^2^ per day [74]; and Moscow Region, where it was 8 (7–10) mg/m^2^ per day [75]. The mean SPM load at the urban station (33 (9.1–75.8) mg/m^2^ per day) was either slightly higher than that in urban areas in some settlements of Russia (the town of Nizhnevartovsk: 10.4 (1–35) mg/m^2^ per day [69]; the town of Tyumen: 20 (5.4–94.3) mg/m^2^ per day [64]; the city of Moscow: 27 (4–213) mg/m^2^ per day [75]) or several times lower than that in urban and urban–industrial areas (the town of Ulan-Ude (Eastern Siberia): 45–966 mg/m^2^ per day [76]; the town of Tomsk (Western Siberia): 63 (16–303) mg/m^2^ per day [77]; the town of Omsk (Western Siberia): 132 (27.8–1007) mg/m^2^ per day [74]; the town of Usol’e-Sibirskoe (Irkutsk Region): up to 250 mg/m^2^ per day in 2021 [38] and 402 (20.3–3421) mg/m^2^ per day in 2008–2019 [78]).

According to [42,73], the SPM load at the urban and suburban stations corresponded to low levels of pollution with dust (up to 250 kg of dust per km^2^).

A significant difference was found between the mean levels of SPM load at the urban and suburban stations (*p* < 0.001) (Table S2), but no significant relationship between SPM loads during 2009–2023 at the urban and suburban stations was found (*p* > 0.05), therefore indicating the variations in the list of SPM sources at these locations during the period of study.

The linear regression analysis using the ln-transformed total volumes of emissions from stationary sources in the Irkutsk Region for the preceding year as the only independent variable suggested that this factor affected the SPM load at the urban location but had no influence on the SPM load at the suburban location (Figure S6). The result of regression analysis suggests different sources of SPM in the urban and suburban locations, probably associated with non-stationary sources including numerous automobile transport, in the case of the town of Irkutsk, and domestic heating using stoves in rare households in winter, in the case of suburban locations, in combination with local and regional atmospheric transport in both cases.

The temporal trend during the period from 2009 to 2023 was studied using a linear regression analysis comparing ln-transformed SPM load values and the years of investigation. No significant increasing or decreasing temporal changes in SPM load were found at either the urban or the suburban stations (Figure S7).

The results of linear and multiple regression analyses using mean temperature, ln-transformed total precipitation volume, total duration of sunshine, and mean humidity for four months with a stable snow cover before snow sampling and these meteorological indexes for the previous twelve months before sampling (from the March of the previous year to the February of the study year) as independent variables indicated no significant factors affecting the SPM load distribution patterns at either the urban or the suburban stations in 2009–2023.

### 3.4. Organochlorine Concentrations in Snow from the Southern Part of the Irkutsk Region and Comparison with Data from Other Locations

The concentrations of OCPs and PCBs in snow at the urban and suburban stations are presented in Table 2, Table 3, Tables S3 and S4. The descriptions of the number of organochlorine compounds analyzed and the frequency of the organochlorine determination are presented in the Supplementary Materials (Table S1 and Text S3).

#### 3.4.1. HCB

The means/medians and ranges of HCB levels in snow at the urban and suburban stations amounted to 0.81/0.65 (0.17–2.00) and 0.57/0.46 (0.06–1.72) ng/L, respectively. In Russia, there are no maximum permissible levels (MPLs) for organochlorine compounds in snow. Taking into account that snowmelt water enters water bodies [11,15] and pollutants in snow water can pose a danger to human and wildlife, we compared the data obtained with the organochlorine MPL in water. The concentrations of HCB in snow from both stations were considerably lower than the Russian MPL for HCB in drinking water, groundwater, and surface water for households and drinking and cultural purposes (0.001 mg/L [79]). HCB levels at two stations investigated were usually higher than HCB levels found in remote locations in mountainous areas in Europe (Alps: 0.004 ng/L [80]; Tatra Mountains: 0.0034–0.0099 ng/L [71]) and Asia (Mt. Everest: 0.033 ng/L [81], as well as in the Arctic (Canada: 0.0006–0.002 ng/L [82]) and Antarctic (0.008 ng/L [83]). This was a result of the regional transport from the Usol’e-Sibirskoe industrial area, where elevated HCB levels had previously been found in the environmental media [22,38].

#### 3.4.2. HCHs and DDTs

The mean/median levels and ranges of the sum of α- and γ-HCH in snow at the urban and suburban stations amounted to 1.42/0.19 (bdl–8.31) and 1.23/0.24 (bdl–5.02) ng/L, respectively. HCHs levels were lower than the Russian MPLs for HCHs in surface water for households and drinking and cultural purposes (0.002 mg/L [79]) and in freshwater water bodies for fishery purposes (0.00001 mg/L [84]). Total HCH levels found in our study were comparable or higher than sums of α- and γ-HCHs found in remote locations in mountainous areas in Europe (Pyrenees: 0.52 ng/L; Alps: 0.49 and 1.1 ng/L [70]; 0.088 ng/L [80]; Tatra Mountains: 0.026–0.075 ng/L [71]). The ratio α/γ-HCH in snow at the urban and suburban stations varied between 0.1 and 2.6, suggesting both technical HCH and lindane as possible sources of HCH here. Taking into account the historical/past application of HCH at closely located agricultural areas [85], the high ability of HCH for atmospheric transport [86], and the bans of technical HCH and lindane application in agriculture in Russia since 1986 and 1990, respectively [87], the presence of HCHs in snow at these stations is a predominant result of the regional atmospheric transport of HCHs evaporated from the agricultural soil surface. The sites of the storage of obsolete pesticides are also additional possible sources of the input of HCH in environmental media at present [88].

The mean/median levels and ranges of the sum of *p*,*p*′-DDT and its metabolites (*p*,*p*′-DDD and *p*,*p*′-DDE) in snow at the urban and suburban stations amounted to 4.05/2.16 (0.24–18) and 5.35/2.88 (0.59–25) ng/L, respectively. Total DDT concentrations in snow collected in this study were much lower than the Russian MPLs for DDTs in surface water for households and drinking and cultural purposes (0.1 mg/L [89]). However, the total DDT concentrations were higher than those found in remote locations in mountainous areas in Europe (Alps: 0.33 ng/L [70] and 0.001 ng/L [85]; Tatra Mountains: 0.073 ng/L [70]) and polar regions (Antarctic: 0.005, 0.015, and 0.024 ng/L [90]). The agricultural application of DDT and pesticide mixtures containing DDT was banned in 1970 [87]. The historical/past DDT application against insects that were carriers of pathogens of vector-borne diseases was previously suggested for the Irkutsk suburban area [91]. The ratio of (DDD + DDE)/DDT was used as an indicator of the past or present release of DDT into the environment [17]. A (DDD + DDE)/DDT value of below 1 indicates a “fresh” supply of DDT. The ratio of (*p*,*p*′-DDD and *p*,*p*′-DDE)/*p*,*p*′-DDT was below 1 in every sample at the urban station and in 77% of samples at the suburban station (Table 1), thus suggesting a continuous entrance of DDT into the environmental media of the southern part of the Irkutsk Region.

#### 3.4.3. PCBs

The mean/median values and ranges of total PCBs in snow at the urban and suburban stations amounted to 81/75 (9.6–196) and 115/71 (8.9–658) ng/L, respectively. The sum of six indicator PCBs (PCB-28, 52, 101, 138, 153, 180) made up about 30–40% of total PCB content (mean/medium (min–max): 27/24 (3.8–66) ng/L at the urban station and 41/24 (3.1–270) ng/L at the suburban station). PCB levels are not normed in surface water for households and drinking and cultural purposes in Russia at present [79]. However, there is an MPL for PCBs in the sum together with DDTs, lindane, aldrin, and other organochlorine toxicants in freshwater water bodies for fishery purposes (0.00001 mg/L [84]). In this case, the MPL was exceeded from 1.5 times in 2009, 2010, and 2019 to 67 times in 2022. PCBs contributed between 44 and 99% to these sums of organochlorine compounds investigated in snow in different years, followed by the DDT share (1–44%). Among other POPs (dioxins and related compounds), the influence of the source located at the Usol’ekhimprom industrial area on the pollution of the southern part of the Irkutsk Region by PCBs was found earlier [21,32].

The highest total PCB concentrations fall into the range of total PCBs in snow from urban locations (Moscow, Russia: ∑PCB_13_ = 280–560 ng/L [92]; Talgar, Kazakhstan: ∑PCB_16_ = 120–800 ng/L [93]). The medians of total PCBs in snow at two Irkutsk stations were similar to the total PCB levels found in urban snow in the city of Almaty (Kazakhstan) (nd–3790 ng/L in 2014–2020 and 9–71 ng/L in 2018–2020 [93]) but were higher than those at urban and industrial locations in Canada (0.7–45 ng/L [94]) and Minneapolis (7.9, 4.6, 1.9 ng/L [6]). The lowest total PCB levels found in our study were higher than those at rural areas in Finland (0.264, 0.285 ng/L [95] and remote areas in the mountains of Europe (Pyrenees: 0.22 ng/L; Alps: 0.73, 2.2 ng/L; Tatra Mountains: 0.2 ng/L [70]) and Asia (Mt. Everest: 0.016 ng/L [81]), as well as those at remote polar regions (Antarctica: 0.138, 0.144, 0.156 ng/L [90]; Canadian Arctic: 0.233–0.788 [82]; Russian Arctic: 0.005 ng/L [96].

#### 3.4.4. PCB-11

PCB-11 is one of the non-Aroclor congeners. The distribution of PCB-11 in the environment is not associated with the production or the application of technical PCB mixtures (Aroclors, etc.). PCB-11 is unintentionally formed during the production of pigments [97]. We analyzed PCB-11 in snow samples collected in 2018–2023. The mean, median, and range values of PCB-11 in snow amounted to 0.74/0.78 (0.05–1.5) and 0.81/0.51 (bdl–2.2) ng/L at the urban and suburban stations, respectively (Tables S3 and S4), which were higher than those found in snow of the polar region (Antarctica (12–142 pg/L [98], 10–34 pg/L [99]). The mean contribution of PCB-11 in the total PCBs made up 0.61%, and it did not exceed 1.5% at both Irkutsk stations. The same contribution of PCB-11 to total PCBs was found in 55 samples from the southern part of the Irkutsk Region in 2021 (0.55 (bdl–2.6)% [38]), as well as in the Arctic snow (0.9–4.5%) [100]. In Antarctica, the contribution of PCB-11 in total PCBs was higher (5.8–22.9%) [99] than that found in the urban area investigated in our study.

### 3.5. Deposition Rates and Daily Deposition Fluxes

The deposition rates and the daily deposition fluxes (DDFs) of HCB, total HCHs, total DDTs, and total PCBs are presented in Table 4.

No significant differences between mean values of deposition rates and daily deposition fluxes of POPs at the urban and suburban stations were detected (Table 4). However, significant strong and medium correlations between POPs in snow at the urban and suburban stations were found (Table 4), therefore indicating similar POP sources at both stations.

The DDFs of ∑HCHs well correlated with the DDFs of ∑DDTs at both stations (*p* < 0.05) (Tables S5 and S6). The DDF of HCB correlated with the DDFs of HCHs and DDTs at the urban station and with PCBs at the suburban station (*p* < 0.05). The DDFs of DDTs were related to the DDFs of total PCBs and PCB homologues at both stations (*p* < 0.05). The combination effect of several sources in the area was assumed. Taking into account the elevated concentrations of PCBs, HCB, and DDTs in the environment of the Usol’e-Sibirskoe industrial area and the town of Usol’e-Sibirskoe [22], and the predominating north–west atmospheric transport in the area [25] (see Section 3.1), the Irkutsk suburban station area was mainly affected by emissions from the Usol’e-Sibirskoe area during the 2009–2023 observation period. There were also additional sources of POP in the urban station area, for example, the application of HCHs in the past at agricultural fields surrounding the town of Irkutsk in the north and west–north directions.

A positive significant correlation was observed between the DDFs of non-Aroclor congener PCB-11 and total PCBs and lower chlorinated PCB homologues and congeners at both stations (*r* = 0.81–0.97, *p* < 0.05). In 2021, a negative correlation between PCB-11 levels and other Aroclor congeners of PCBs was found in snow collected in the southern part of the Irkutsk Region [38], where several locations of PCB emission are located. The positive correlation obtained for these two stations during 2018–2023 could be associated with the source of emission of both the non-Aroclor congener PCB-11 and other Aroclor/Sovol congeners of PCBs located in the same territory as well as with a similar degree of operation of the source during the study period. The highest PCB-11 levels in snow of the Irkutsk Region were previously found in snow sampled on the Usol’e-Sibirskoe industrial area [38], where a paint factory, which could be an unintentional source of PCB-11, was also located in the past [97].

### 3.6. Temporal Trend of Daily Deposition Fluxes of PCBs and OCPs and the Effect of Technogenic Activity in Southern Part of Irkutsk Region in 2009–2023

Temporal trends of daily deposition fluxes (DDFs) during the period from 2009 to 2023 were investigated using a linear regression analysis comparing ln-transformed DDF values of POPs analyzed every year and years of investigation. Significant results of the analysis are presented in Table S7.

#### 3.6.1. DDFs of HCHs and DDTs

Significant changes were found for DDFs of α-HCH, γ-HCH, and the sum of HCHs in the snow at both Irkutsk stations (Table S7).

The highest DDFs of total HCHs amounted to 4.9 and 4.8 ng/m^2^ per day at the urban and suburban stations, respectively in 2012, followed by 2.6 ng/m^2^ per day in 2009 and 2.1 ng/m^2^ per day in 2013 at the suburban station and 2.5 ng/m^2^ per day in 2013 at the urban station. From 2018, HCH levels were usually below the MDL at both Irkutsk stations. The changes in total HCH DDF correlated well with the interannual variations in HCHs in air over this area in 2009–2017 [91].

Significant decreasing trends were also observed for DDFs of *p*,*p*′-DDD in snow at the urban station and DDFs for *p*,*p*′-DDT and ∑*p*,*p*′-DDX at the suburban station. The highest levels of DDF for ∑*p*,*p*′-DDX were found in 2012 at the urban station (10.8 ng/m^2^ per day) and in 2009 at the suburban station (16 ng/m^2^ per day). (DDD + DDE)/DDT ratios increased significantly (*p* < 0.01) during the studied period from 0.10 at the urban station in 2011 and 0.03–0.09 at the suburban station in 2009–2010 up to 0.61–0.94 and 0.74–1.26 at the urban and suburban stations, respectively in 2020–2023 (Figure S8). A single elevation of (DDD + DDE)/DDT ratios up to 2.14 at the suburban station was obtained in 2012. The variations in the DDFs of DDT and its metabolites and their relative compositions during 2009–2023 indicate the continuing decreasing trend of this pesticide’s abundance in the environment of Eastern Siberia, which was detected previously [38,91].

#### 3.6.2. DDFs of HCB and PCBs

No linear changes were found for the DDF of HCB at both stations in 2009–2023. The changes in HCB DDFs are likely polynomial with minima in 2010–2011 and 2018–2019 and maxima in 2012–2013 and 2022–2023 (Figure S9). The highest levels of HCB DDF were observed in the periods when Usol’ekhimprom finished its operation in 2012 and when the workshop buildings were dismantled at the Usol’ekhimprom industrial area in 2021–2022. In 2009–2017, no changes in the average annual levels of HCB in air were detected, but an increasing trend of the average HCB levels in air of this area was obtained for every two-month period [91].

**Figure 2:**
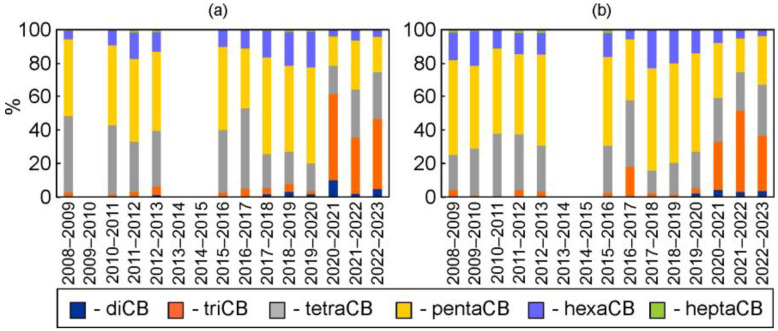
Relative PCB homological compositions in snow at the urban (**a**) and suburban (**b**) stations in 2009–2023.

On the other hand, significant temporal trends were also found for DDFs of PCB-28, PCB-180, and triCB in the snow at both Irkutsk stations, as well as for DDFs of PCB-70 + 76 and PCB-170 at the suburban station (Table S7). Changes in lower chlorinated congeners and homologies of PCBs represented an increasing trend. On the contrary, the changes in higher chlorinated congeners of PCBs represented a decreasing trend. Significant changes in relative homological and congener compositions of PCBs were also detected (Figure 2). For example, the portions of triCB in total PCBs increased from 1.1–3.0% and 0.2–4.4% in 2009–2012 to 33–52% and 29–49% in 2021–2023 at the urban and suburban stations, respectively (*p* < 0.05). The portions of hexaCB decreased from 11–21% in 2009–2012 to 3.5–7.8% in 2021–2023 in snow at the suburban station (*p* < 0.05).

Taking into account certain periods with suspected different degrees of organochlorine emissions from the industrial area of Usol’ekhimprom and elevated levels of PCBs, and HCB detected earlier at the industrial area of Usol’e-Sibirskoe [21,22], the mean values of the daily deposition fluxes (DDF) of PCBs and HCB were compared for the three periods including 2008–2012 (operational activity of the Usol’ekhimprom enterprise with the control of the waste storage locations by this enterprise), 2013–2020 (uncontrolled waste storage and the introduction of municipal and regional states of emergency due to the threat of chemical pollution), and 2021–2023 (activities for eliminating the accumulated environmental damage in the Usol’ekhimprom industrial area, started in the fall of 2020).

**Figure 3:**
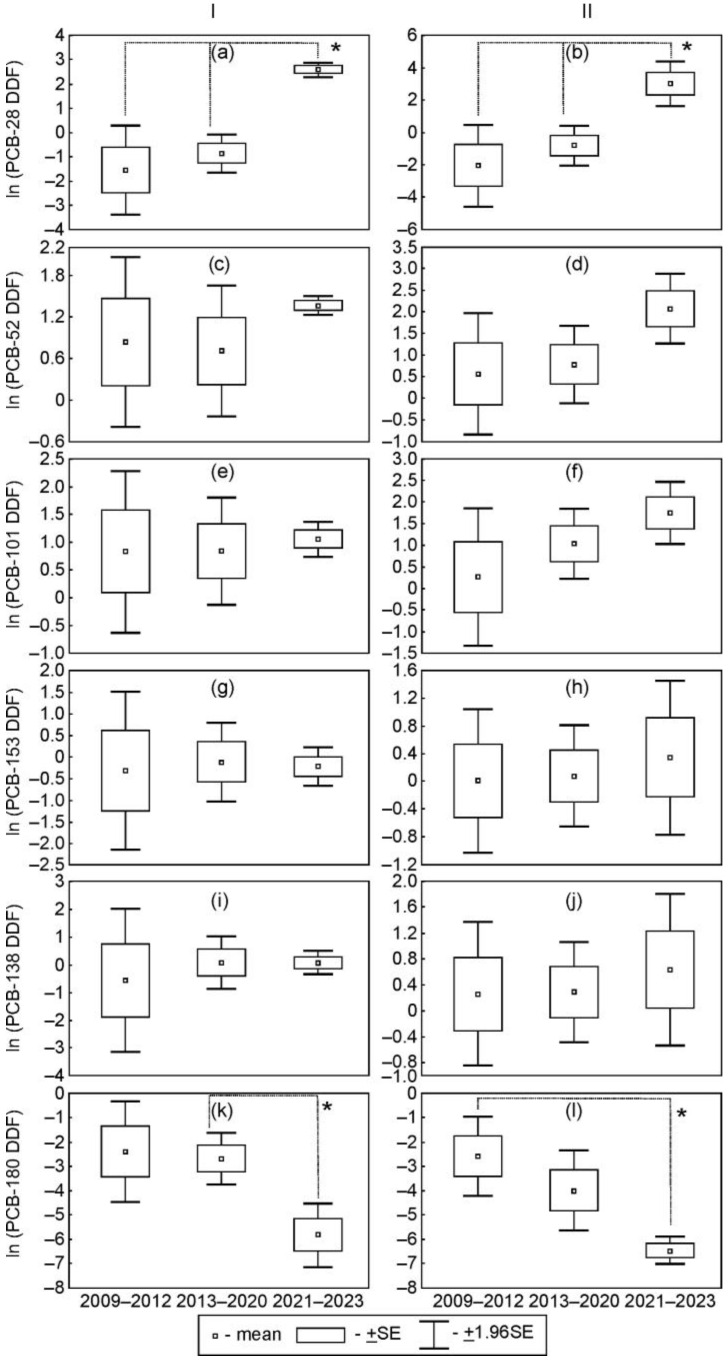
The comparison of mean values of daily deposition fluxes (DDF) of indicator PCB congeners at the urban (**I**) and suburban (**II**) stations in three periods of activities at the Usol’ekhimprom industrial area (Ln (ng/m^2^ per day); *—*p* < 0.05).

The changes in mean daily deposition fluxes (DDFs) for both the total amounts of six indicator PCBs and PCB homologues and individual congeners of PCBs in 2008–2012 and 2013–2020 were insignificant at both stations (Figure 3).

**Figure 4:**
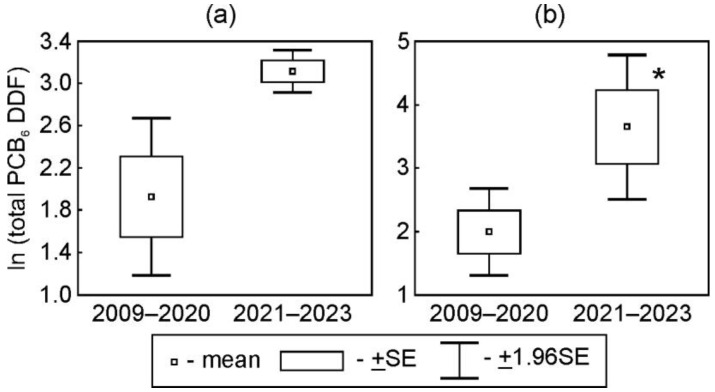
Comparison of mean values of daily deposition fluxes of total amounts of indicator PCBs at the urban (**a**) and suburban (**b**) stations in periods of different activities at the Usol’ekhimprom industrial area (Ln (ng/m^2^ per day); *—*p* < 0.05).

The variations in PCB DDFs between the first + second (2009–2020) and third (2021–2023) periods were dependent on the chlorination level. The mean values of DDFs of PCB-28 and diCB and triCB in 2021–2023 were significantly higher than the DDFs during previous periods at both stations (*p* < 0.05) (Figure 3a,b and Figure S10a–d). The DDFs of PCB-180 in 2021–2023 were significantly lower than those in 2013–2020 at the urban station and those in 2009–2012 at the suburban station (*p* < 0.05) (Figure 3k,l). The mean values of DDFs of the total amounts of six indicator PCBs in 2021–2023 were higher than those observed in 2009–2020 at the urban (*p* > 0.05) and suburban (*p* < 0.05) stations (Figure 4).

Such temporal variations in PCB DDFs suggested a change in emissions from the Usolekhimprom industrial area at least since the fall of 2020, when the efforts to eliminate the accumulated environmental damage in the Usol’ekhimprom industrial area started with the dismantling of the above-ground part of the mercury electrolysis workshop building and the pumping out and conservation of two wells for the deep burial of epichlorohydrin waste. In 2021–2023, those efforts were continued and included the dismantling of other under- and above-ground constructions of workshop buildings and the liquidation and conservation of other wells for the deep burial of other waste in 2021–2023 [34].

**Figure 5:**
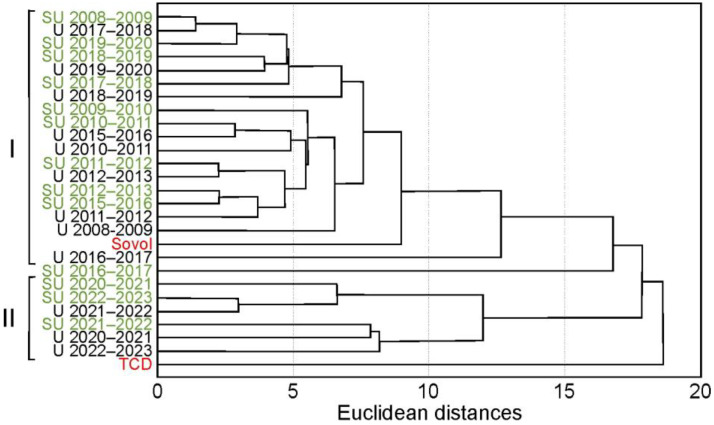
The grouping of snow sampled at the urban (U) and suburban (SU highlighted in green) stations and PCB technical mixtures Sovol and TCD [101] (highlighted in red) by relative PCB homological patterns using the cluster method.

The change in the relative homological PCB pattern in the winters of 2020–2023 was also confirmed with the cluster method (Figure 5), which formed two groups of the snow samples collected at both stations in 2009–2023. The first group included snow sampled in the Februarys of 2009–2020, which was characterized by the domination of pentaCBs, followed by tetraCBs and hexaCBs, and was comparable to the PCB homological pattern in soil samples from the Usol’e-Sibirskoe area [22] and in the technical PCB mixture Sovol [101] (Figure 5 and Figure S11). Snow samples collected at the ends of winters in 2020–2021, 2021–2022, and 2022–2023 were included in the second group (Figure 5). In this group, triCBs dominated amongst PCB homologues, followed by tetraCBs and pentaCBs (Figure 2). The results from the cluster method are presented in Text S4.

The change in congener PCB patterns in snow is presented in Table S8, which provides the list of the first ten most abundant PCB congeners. In 2008–2020, pentachlorinated congeners PCB-101/90, 110, 99, and 118; tetrachlorinated congeners PCB-44, 52, and 66; and hexachlorinated congeners PCB-138 and 153 contributed more than 5% each to the total PCB concentration. Since the winter of 2020–2021, the sum of PCB-28 and PCB-31 contributed up to 31–52% and 29–46% to the total PCB concentrations at the urban and suburban stations, respectively, in comparison with 0.6–4.8% in previous winters. The description of Table S8 is presented in more detail in the Supplementary Materials (Text S4). The same changes in PCB congener composition were found in the majority of snow samples collected in the southern part of the Irkutsk Region in the winter of 2020–2021 [38]. We considered then that the increase in the emissions of lower chlorinated compounds in snow sampled in the southern part of the Irkutsk Region was a result of the application of water curtains during the dismantling of buildings of Usol’ekhimprom to avoid the aerial transport of particles containing mercury [34,38], but this technique was used only during the dismantling of the mercury electrolysis workshop building in the fall of 2020 [34].

So, the shift of PCB homological and congener patterns to lower chlorinated PCBs may have resulted from total activities at the Usol’ekhimprom industrial area since the fall of 2020. The disturbance of the long-term industrial soil surface could have contributed to the contact of more PCB-polluted underlying layers of soil with air and the evaporation of mainly PCBs with a low chlorine content into the air. In addition, the destruction of the workshop buildings may have resulted in the massive release of PCB from building structures into the environmental media. It is known that PCBs were used as plasticizers in the production of building materials including paints [102].

**Figure 6:**
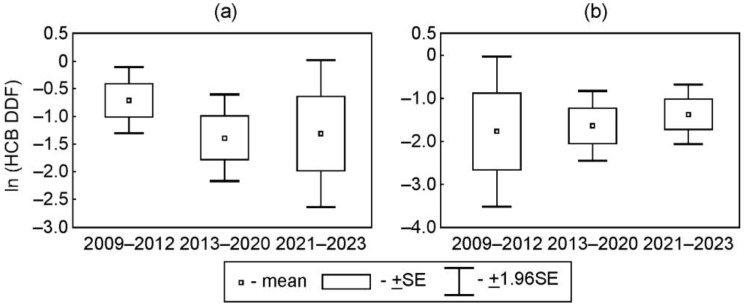
The comparison of mean values of daily deposition fluxes of HCB at the urban (**a**) and suburban (**b**) stations in periods of different activities at the Usol’ekhimprom industrial area (Ln (ng/m^2^ per day)).

The mean values of the DDFs of HCB in 2013–2020 and 2021–2023 were either slightly lower than those in 2009–2012 at the urban station or comparable to those in 2009–2012 at the suburban station, thus suggesting no considerable variations in the entry of HCB into the environment of the southern part of the Irkutsk Region in winter during the study period of 2009–2023 (Figure 6).

Thus, the changing of technogenic activity in the Usol’ekhimprom industrial area during the study period did not result in a noticeable effect on the HCB daily deposition flux in the southern part of the Irkutsk Region. However, the activities in the industrial area of Usol’ekhimprom, which began in the fall of 2020 and were related to the elimination of the accumulated environmental damage, had a significant impact on the concentrations of lower chlorinated PCBs in the snow cover and daily deposition fluxes of these PCBs with wet precipitation, at least in winter.

### 3.7. The Relationship of Daily Deposition Fluxes (DDFs) of POPs with Suspended Particulate Matter (SPM) Load and Total Emissions from Stationary Sources in Irkutsk Region

The relationship between daily deposition fluxes (DDFs) of PCBs, DDTs, HCHs, and HCB and SPM loads in 2009–2023 was investigated using multiple regression analysis using ln-transformed SPM loads at both Irkutsk stations and ln-transformed values of emissions from stationary sources for previous years as independent variables. This regression analysis showed no effect of SMP loads and emissions from stationary sources on the DDFs of POPs studied at both Irkutsk stations in 2009–2023. However, a significant positive relationship between the distributions of POPs and SPM in snow was previously found during simultaneous snow sampling in several dozen locations in the southern part of the Irkutsk Region in 1994–1996 and 2021 [38].

### 3.8. Temperature, Precipitation, Air Humidity, and Sunshine Duration Effect on Daily Deposition Fluxes (DDFs) of PCBs and OCPs

Multiple regression analysis using mean air temperature, ln-transformed sums of precipitation volumes, ln-transformed mean air humidity, and ln-transformed sum of sunshine duration for four months with stable snow cover (November and December of the previous year; January and February of the study year) (Table S9) as independent variables indicated that the significant factor accounting for most of the variance included air humidity as the sole independent variable (HCB, *p*,*p*′-DDT, total *p*,*p*′-DDX, and PCB-87 at the urban station; PCB-170 at the suburban station; and α-HCH, γ-HCH, α + γ-HCH, and PCB-66 at both stations), or in combination with temperature (PCB-87 at the suburban station), precipitation volume (*p*,*p*′-DDT at the suburban station), or sunshine duration (PCB-149 at the suburban station), precipitation volume + sunshine duration (PCB-47 at the urban station), or precipitation volume + sunshine duration + temperature (PCB-8, 47, and 169 and diCBs at the suburban station). Temperature was the key factor influencing PCB-52, 49, 44, 101, 110, and 118 and total PCBs, triCBs, tetraCBs, and pentaCBs at the suburban station and PCB-169 at the urban station, being the sole independent variable (Table S9). The increases in air humidity and air temperature resulted in increases in the DDFs of PCBs and OCPs. The increases in the precipitation volume and/or sunshine duration negatively affected DDF values. The air humidity, unlike the precipitation volume, affects the DDF of OCPs and PCBs, and may be the result of the variability in the precipitation volume over an area. At the same time, the air humidity is a more spatially stable index than the precipitation volume. Air humidity and temperature are significant factors in the formation of precipitation [1]. In addition, the air humidity can affect the adsorption of organic pollutants by solid particles or droplets of freezing water and contributes to the dry and wet deposition of POPs. The negative effect of sunshine duration can result from photochemical degradation of POPs in snow [103].

Due to considerable variations (up to 15 times; see Section 3.2) in precipitation volumes during winter months, the relationships between the DDFs of POPs and monthly means precipitation volumes were studied using multiple regression analysis using mean values of precipitation volume for every month with a stable snow cover before snow sampling (November and December of the previous year; January and February of the study year) (Table S10). The precipitation volume in February was a key factor affecting HCB, DDE, and most of the PCB congeners in the suburban location, as opposite to the urban location (Table S10). The organic pollutants that entered the snow cover with snowfall in February on the eve of snow likely had no time to degrade under the influence of various factors including ultraviolet light [103], singlet molecular oxygen, and other factors [104]. The absence of a temporary effect of the precipitation volume during winter months on DDFs of POPs at the urban location could have resulted from higher particulate-matter pollution in the urban area compared to suburban area, as presented in Section 3.3. Particulates can adsorb POPs and additionally enter the snow cover together with POPs as a result of dry deposition from the atmosphere [2].

## 4. Conclusions

This study discussed long-term variations in the distribution of suspended particulate matter (SPM) and persistent organic pollutants (POPs) in snow cover at two stations in the Irkutsk Region, Eastern Siberia, Russia, as well as the influence of meteorological factors and technogenic activities at the former industrial area of an organochlorine company on these fluctuations. SPM loads at both locations corresponded to low levels of pollution. The individual concentrations of HCB, HCHs, and ∑*p*,*p*′-DDX in snow from both stations were considerably lower than the Russian maximum permissible levels (MPLs) for these compounds in water for households and drinking and cultural purposes as well as the MPLs in water for fishery purposes. However, the sums of DDTs, lindane, and other organochlorine toxicants including PCB were 1.5–67 times higher than the MPL of the sums in freshwater water bodies for fishery purposes.

The SPM levels and SPM loads were significantly higher at the urban locality than at the suburban one. However, the concentrations, deposition rates, and daily deposition fluxes of POPs were comparable and well correlated between these stations.

The contents of PCBs and OCPs in snow show either decreasing (HCHs and DDTs, higher chlorinated PCBs) or increasing (low chlorinated PCBs) trends or present insignificant fluctuations (SPM, HCB) only for winter. Long-term studies of POPs in the atmospheric air, combined with investigations of POPs in snow and soil studies at the same locations, are required to understand the fate of the compounds in all seasons of a year, as well as interannual variations in the POPs contents in the environment.

It was established that the recultivation activity at the industrial area of the former organochlorine enterprise changed the PCB levels and relative compositions of PCB congeners and PCB homologues in snow.

Possible effects of air temperature, precipitation volumes, air humidity, and sunshine duration on the distribution of SPM loads and POP DDFs were investigated as well. No relationship was found between SPM loads and meteorological factors. In case of POPs, the air humidity and temperature influenced the DDFs of POPs either as sole independent variables or in combination with other meteorological factors.

## Figures and Tables

**Table 1:** The characteristics of the snow cover at the time of sampling at the urban and suburban stations and concentrations, deposition rates, and loads of suspended particulate matter (SPM) in snow in 2009–2023.

Characteristic	N	Mean	Median	Min	Max	SD *	SE **
	urban station						
height, cm	12	32.7	31	23	42	5.5	1.6
density, g/cm^3^	12	0.16	0.17	0.11	0.20	0.02	0.01
SWE, mm	12	54.6	56.4	25.9	72.0	13.4	3.9
time of snowpack, days	12	108	107	91	129	12	4
SPM, mg/L	12	66	51	24	137	37	11
SPM deposition rate, mg/m^2^	12	3515	2621	1176	7898	2099	606
SPM load, mg/m^2^ per day	12	33	27	9	76	20	5.9
	suburban station						
height, cm	13	40.1	38	21	67	10.4	2.9
density, g/cm^3^	13	0.15	0.15	0.10	0.19	0.03	0.01
SWE, mm	13	61.7	62.2	21.0	115	22.4	6.2
time of snowpack, days	13	107	104	91	129	12	3
SPM, mg/L	13	12.8	12.8	1.8	38	9.1	2.5
SPM deposition rate, mg/m^2^	13	693	786	115	1273	362	100
SPM load, mg/m^2^ per day	13	6.5	6.7	1.3	11.6	3.4	0.95

*—standard deviation, **—standard error.

**Table 2:** The mean, median, range (min–max), standard deviation (SD), and standard error (SE) values of organochlorine levels and ratios of HCHs and DDTs in snow found in 2009–2023 at the urban station (ng/L).

Compound	N	Mean	Median	Min	Max	SD	SE
HCB	12	0.81	0.65	0.17	2.00	0.63	0.18
α-HCH	12	0.78	0.05	BDL	4.33	1.35	0.39
γ-HCH	12	0.65	0.14	BDL	3.97	1.21	0.35
α + γ-HCH	12	1.42	0.19	BDL	8.31	2.56	0.74
α/γ-HCH	12	1.3	1.3	0.5	1.9	0.5	0.2
*p*,*p*′-DDT	12	3.18	1.20	BDL	16	4.71	1.36
*p*,*p*′-DDE	12	0.79	0.44	0.14	2.05	0.70	0.20
*p*,*p*′-DDD	11	0.08	0.02	BDL	0.42	0.16	0.05
*o*,*p*′-DDT	6	0.30	0.02	BDL	1.41	0.56	0.23
*o*,*p*′-DDE	6	BDL	BDL	BDL	BDL	-	-
*o*,*p*′-DDD	6	BDL	BDL	BDL	BDL	-	-
∑*p*,*p*′-DDX	12	4.05	2.16	0.24	18	5.40	1.56
(*p*,*p*′-DDE+ *p*,*p*′-DDD)/*p*,*p*′-DDT	12	0.45	0.37	0.10	0.94	0.29	0.09
PCB-28	12	7.24	1.51	0.10	29	11.3	3.25
PCB-52	12	6.53	6.03	0.94	13.4	4.32	1.25
PCB-101 or 101 + 90	12	7.02	4.13	1.21	22	6.27	1.81
PCB-153	12	2.74	1.31	0.35	11	3.24	0.93
PCB-138	12	3.56	1.56	0.11	16	4.57	1.32
PCB-180	12	0.26	0.07	BDL	1.26	0.40	0.12
∑PCB_all_	12	81	75	9.6	196	62	18
∑PCB_6_	12	27	24	3.8	66	21	6.1
diCB	9	2.22	0.68	0.07	9.38	2.97	0.99
triCB	12	13	3.16	0.22	49	19	5.48
tetraCB	12	21	20	2.26	50	15	4.41
pentaCB	12	33	17	4.42	110	33	9.55
hexaCB	12	9.58	4.46	0.46	41	12	3.46
heptaCB	12	0.55	0.11	0.03	2.39	0.86	0.25

**Table 3:** The mean, median, range (min–max), standard deviation (SD), and standard error (SE) values of organochlorine levels and ratios of HCHs and DDTs in snow found in 2009–2023 at the suburban station (ng/L).

Compound	N	Mean	Median	Min	Max	SD	SE
HCB	13	0.57	0.46	0.06	1.72	0.50	0.14
α-HCH	13	0.66	0.03	BDL	2.87	1.03	0.29
γ-HCH	13	0.56	0.11	BDL	2.71	0.93	0.26
α + γ-HCH	13	1.23	0.24	BDL	5.02	1.89	0.53
α/γ-HCH	13	1.4	1.0	0.1	2.6	0.9	0.3
*p*,*p*′-DDT	13	4.34	2.22	0.42	23	6.41	1.78
*p*,*p*′-DDE	13	0.94	0.56	0.08	3.51	1.06	0.29
*p*,*p*′-DDD	10	0.09	0.004	BDL	0.45	0.19	0.06
*o*,*p*′-DDT	6	0.23	0.09	BDL	0.95	0.37	0.15
*o*,*p*′-DDE	6	BDL	BDL	BDL	BDL	-	-
*o*,*p*′-DDD	6	0.01	0.004	BDL	0.06	0.02	0.01
∑*p*,*p*′-DDX	13	5.35	2.88	0.59	25	6.69	1.85
(*p*,*p*′-DDE+ *p*,*p*′-DDD)/*p*,*p*′-DDT	13	0.59	0.31	0.03	2.14	0.62	0.17
PCB-28	13	17	1.15	0.08	183	50	14
PCB-52	13	8.87	5.86	0.78	39	11	2.96
PCB-101 or 101 + 90	13	8.02	5.90	0.45	26	8.32	2.31
PCB-153	13	2.92	1.81	0.71	9.16	2.78	0.77
PCB-138	13	3.84	2.51	0.90	13	3.76	1.04
PCB-180	13	0.12	0.05	BDL	0.61	0.17	0.05
∑PCB_all_	13	115	71	8.9	658	172	48
∑PCB_6_	13	41	24	3.1	270	71	19
diCB	9	3.21	0.42	0.05	18	5.87	1.96
triCB	13	31	2.20	0.04	319	87	24
tetraCB	13	30	20	2.50	154	40	11
pentaCB	13	40	25	4.45	131	41	11
hexaCB	13	9.91	5.86	1.84	32	9.86	2.73
heptaCB	13	0.24	0.06	0.01	1.31	0.37	0.10

**Table 4:** Deposition rates (ng/m^2^) and daily deposition flux (DDF) (ng/m^2^ per day) of POPs at two stations in Irkutsk Region in 2009–2023 (mean/median and range) (R—coefficient of correlation, *p*—reliability).

	Urban Station	Relationship of Values in Urban and Suburban Stations		Suburban Stations
		R	*p*	
	Deposition rates (ng/m^2^)			
HCB	47/33 (6–126)	0.65	<0.05	39/32 (3.8–199)
α-HCH + γ-HCH	94/9 (bdl–598)	0.96	<0.001	96/15 (bdl–578)
∑*p*,*p*′-DDX	250/105 (6–1310)	0.71	<0.05	325/210 (18–1760)
∑PCBs	41/40 (3–99)	0.85	<0.001	60/40 (4–296)
∑6 indicator PCBs	14/13 (1–29)	0.83	<0.001	22/11 (1.4–123)
	DDF (ng/m^2^ per day)			
HCB	0.43/0.30 (0.06–1.08)	0.60	<0.05	0.34/0.30 (0.04–1.64)
α-HCH + γ-HCH	0.80/0.07 (bdl–4.94)	0.96	<0.001	0.81/0.14 (bdl–4.78)
∑*p*,*p*′-DDX	2.22/1.03 (0.06–11)	0.70	<0.05	3.02/1.74 (0.18–16)
∑PCBs	41/40 (3–99)	0.82	<0.001	60/40 (4–296)
∑6 indicator PCBs	14/13 (1–29)	0.83	<0.01	22/11 (1.4–123)

## Data Availability

The data presented in this study will be available on request from the corresponding author.

## Supplementary Materials

The following supporting information can be downloaded at: https://www.mdpi.com/article/10.3390/toxics12010011/s1, Figure S1. Two-day forecast trajectory from land source located in the Usol’ekhimprom industrial area on 28 January 2021 (a) and 29 January 2021 (b); Figure S2. Two-day backward air trajectory at the urban (a,c,e,g) and suburban (b,d,f,h) stations situated 500 (a,b), 800 (c,d), 1000 (e,f), and 1500 (g,h) m above ground level on 29 January 2021; Figure S3. Two-day backward air trajectory at the urban and suburban stations situated 800 m above ground level in days with highest precipitation volumes in the winter of 2020–2021; Figure S4. Climatological Standard (1991–2020) [62] and Reference (1961–1990) [63] Normals and average, minimal, and maximal average monthly values of temperature (a, °C) [43], precipitation volume (b, mm) [44], and duration of sunshine (c, hours) [46] in period of snow study (2009–2023); Figure S5. The snow water equivalent (SWE) variations at the urban (1) and suburban (2) stations, as well as number of days with stable snow cover before snow sampling (3) in winter in 2008–2023; Figure S6. Relationship between the ln-transformed levels of suspended particulate matter (SPM) load at the urban (1) and suburban (2) stations and the ln-transformed total volume of emissions from stationary sources in Irkutsk Region; Figure S7. The load of suspended particulate matter (SPM) at the urban (1) and suburban (2) stations in winter in 2008–2023; Figure S8. Temporal trend of the ratios of DDT and its metabolites in snow at the urban (1) and suburban (2) stations in 2009–2023; Figure S9. Temporal variations in DDFs of HCB in snow at the urban (1) and suburban (2) stations in 2009–2023; Figure S10. The comparison of mean values of daily deposition fluxes (DDF) of PCB homologues in snow at the urban (I) and suburban (II) stations in periods with different operational activities at the Usol’ekhimprom industrial area (Ln (ng/m^2^ per day); *—*p* < 0.05); Figure S11. The relative PCB homological compositions in PCB technical mixtures of Sovol and Trichlorodiphenyl (TCD) [101]; Table S1. The list of PCB congeners and their groups and OCPs analyzed in snow samples, instrumental methods, and types of snow sampling companies (compounds highlighted in red were detected below MDLs at both stations in the given year); Table S2. The significance of differences (*t*-test) and coefficients of correlation and the significance of differences and correlations between values obtained at the urban and suburban stations (“-”—*p* > 0.05); Table S3. The mean, median, range (min–max), standard deviation (SD), and standard error (SE) values of levels of PCB congeners in snow found in 2009–2023 at the urban station (ng/L); Table S4. The mean, median, range (min–max), standard deviation (SD), and standard error (SE) values of levels of PCB congeners in snow found in 2009–2023 at the suburban station (ng/L); Table S5. The significant (*p* < 0.05) correlation coefficients of DDFs of POPs in snow at the urban station (“-”—*p* > 0.05); Table S6. The significant (*p* < 0.05) correlation coefficients of DDFs of POPs in snow at the suburban station (“-”—*p* > 0.05); Table S7. Results of linear regression analysis comparing ln-transformed DDFs of POP values and years of investigation in 2009–2023 (* *p* < 0.05, ** *p* < 0.01, *** *p* < 0.001, «-» *p* > 0.05); Table S8. The list of the first ten abundant PCB congeners and their groups in snow from max to min contribution to total PCB levels in 2009–2023 at the urban (U) and suburban (S) stations (diCB highlighted in orange, triCB highlighted in yellow, tetraCB highlighted in green, pentaCB highlighted in blue, hexaCB highlighted in red; U—urban, S—suburban); Table S9. Results of the multiple linear regression analysis comparing the average temperature of air, ln-transformed total values of precipitation, ln-transformed average air humidity, and ln-transformed total sunshine duration for 4 months (November + December + January + February) during the periods of study in 2008–2023 and ln-transformed values of POP DDFs (* *p* < 0.05, ** *p* < 0.01, *** *p* < 0.001, “-”, *p* > 0.05); Table S10. Results of the multiple linear regression analysis comparing the ln-transformed values of the precipitation volumes for November and December of previous year and January and February of study year in 2008–2023 and ln-transformed values of POP DDFs (* *p* < 0.05, ** *p* < 0.01, “-”, *p* > 0.05); Text S1. Backward and Forecast Air Trajectories; Text S2. Characteristics of snow cover at the time of sampling and some meteorological indexes in 2009–2023; Text S3. The set of organochlorines and the frequency determination; Text S4. The change in the relative homological and congener PCB patterns in snow samples.
