# Supplementary material for: Persistent Organic Pollutants and Suspended Particulate Matter in Snow of Eastern Siberia in 2009–2023: Temporal Trends and Effects of Meteorological Factors and Recultivation Activities at Former Industrial Area
Source: Toxics. 2023 Dec 22;12(1):11. doi: 10.3390/toxics12010011 (PMC10819055; doi:10.3390/toxics12010011)
Supplement: Supplementary file 1 [file toxics-12-00011-s001.zip › toxics-2762260-supplementary.pdf]

toxics

MDPI

Article

Persistent Organic Pollutants and Suspended Particulate Matter in Snow of Eastern Siberia in 2009–2023: Temporal Trends and Effects of Meteorological Factors and Recultivation Activities at Former Industrial Area

Elena A. Mamontova * and Alexander A. Mamontov

Vinogradov Institute of Geochemistry SB RAS, Irkutsk 664033, Russia; mamontov@igc.irk.ru
* Correspondence: elenam@igc.irk.ru

Supplementary data

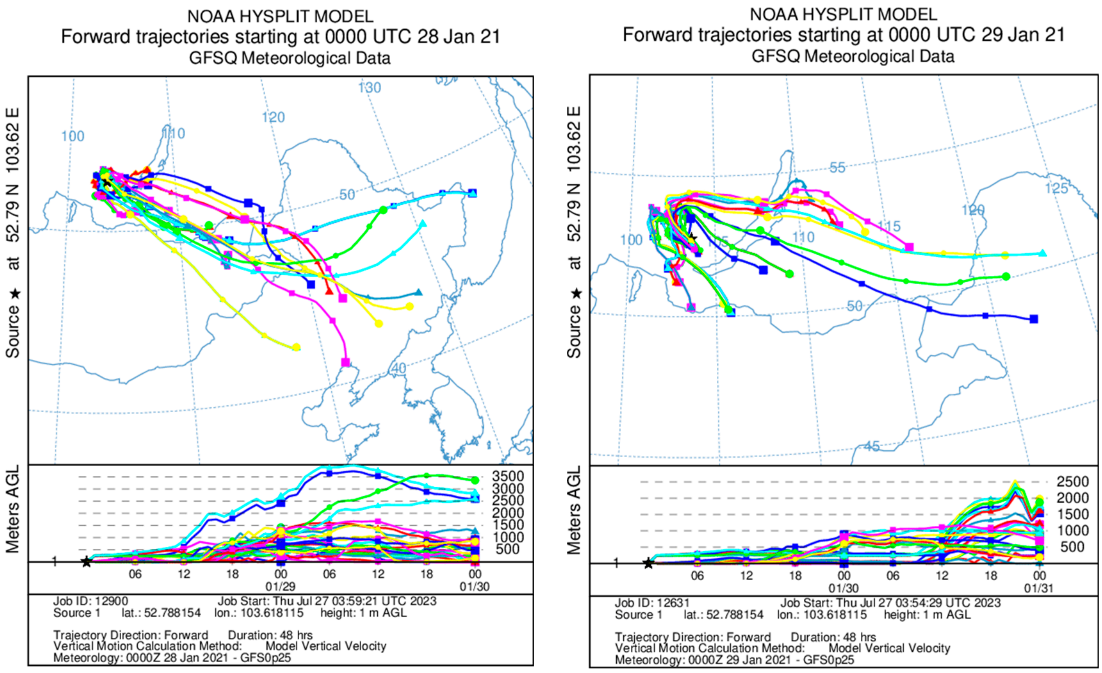

(**a**) (**b**)

Figure S1. Two-day forecast trajectory from land source located in the Usol'ekhimprom industrial area on 28 January, 2021 (a) and 29 January, 2021 (b).

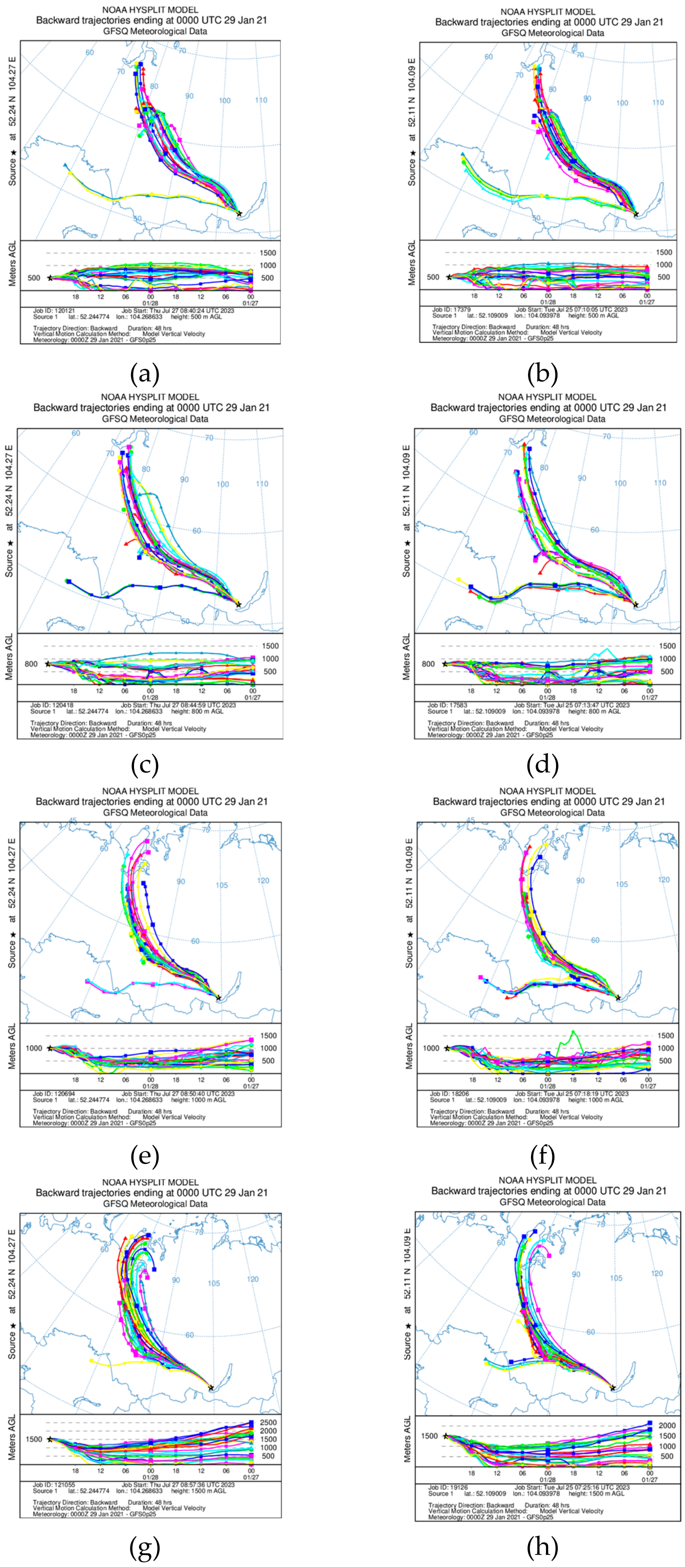

Figure S2. Two-day backward air trajectory at the urban (**a**,**c**,**e**,**g**) and suburban (**b**,**d**,**f**,**h**) stations situated 500 (**a**,**b**), 800 (**c**,**d**), 1000 (**e**,**f**), and 1500 (**g**,**h**) m above ground level in 29 January, 2021.

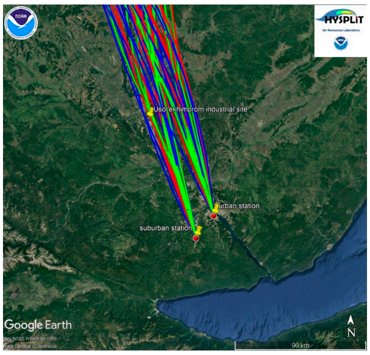

19 November 2020

Precipitation volume—3.1 mm

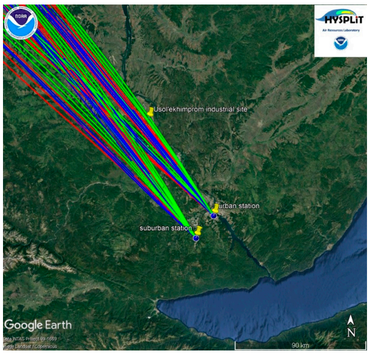

30 November 2020

Precipitation volume—2.5 mm

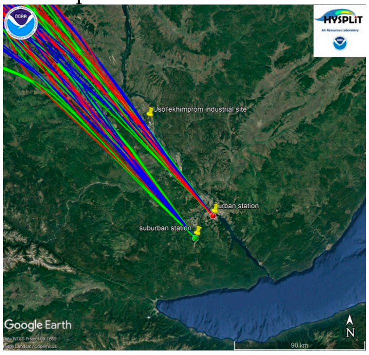

7 January 2021

Precipitation volume—3.7 mm

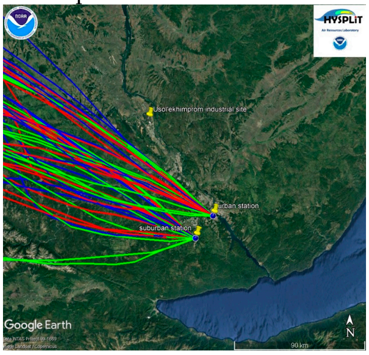

17 January 2021

Precipitation volume—4.3 mm

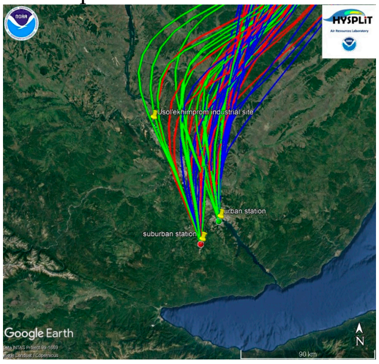

25 January 2021

Precipitation volume—3.1 mm

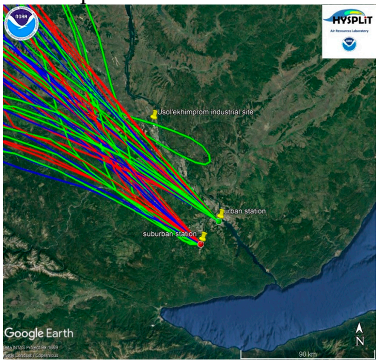

29 January 2021

Precipitation volume—5.4 mm

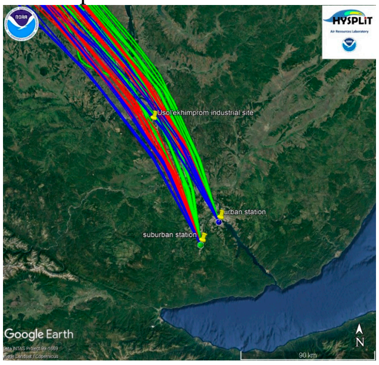

5 February 2021

Precipitation volume—3.4 mm

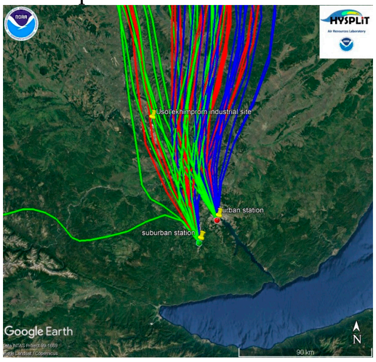

10 February 2021

Precipitation volume—4.4 mm

Figure S3. Two-day backward air trajectory at the urban and suburban stations situated 800 m above ground level in days with highest precipitation volumes in the winter of 2020–2021.

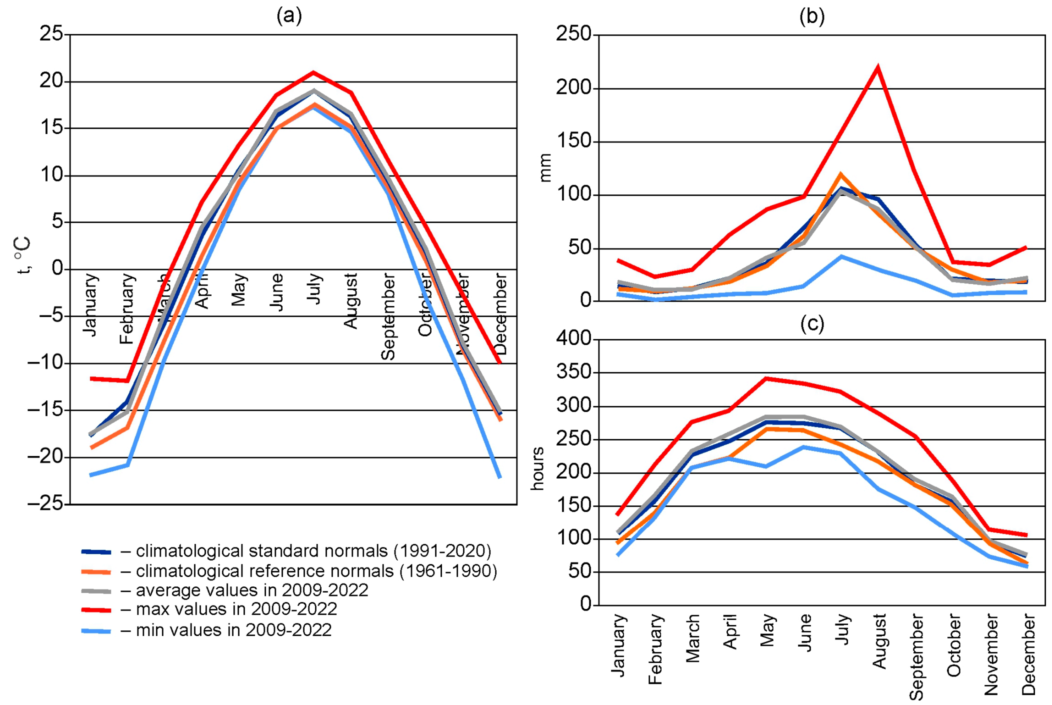

Figure S4. Climatological Standard (1991–2020) [62] and Reference (1961–1990) [63] Normals and average, minimal, and maximal average monthly values of temperature (a, °C) [43], precipitation volume (b, mm) [44], and duration of sunshine (c, hours) [46] in period of snow study (2009–2023).

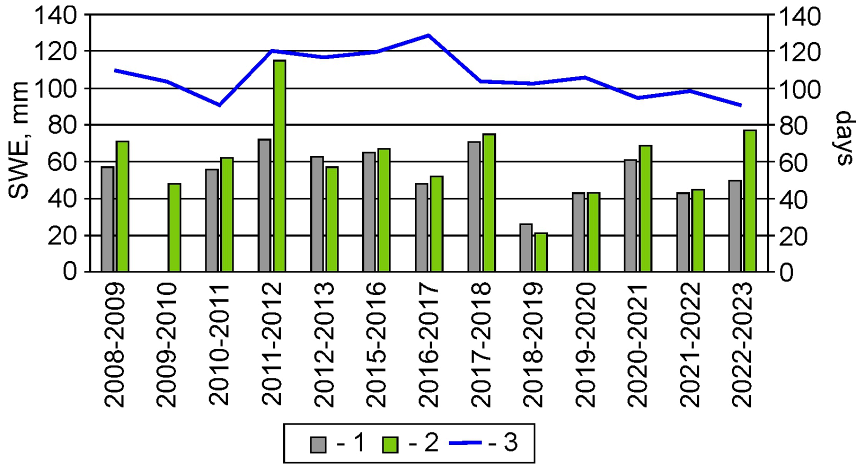

Figure S5. The snow water equivalent (SWE) variations at the urban (1) and suburban (2) stations, as well as number of days with stable snow cover before snow sampling (3) in winter in 2008–2023.

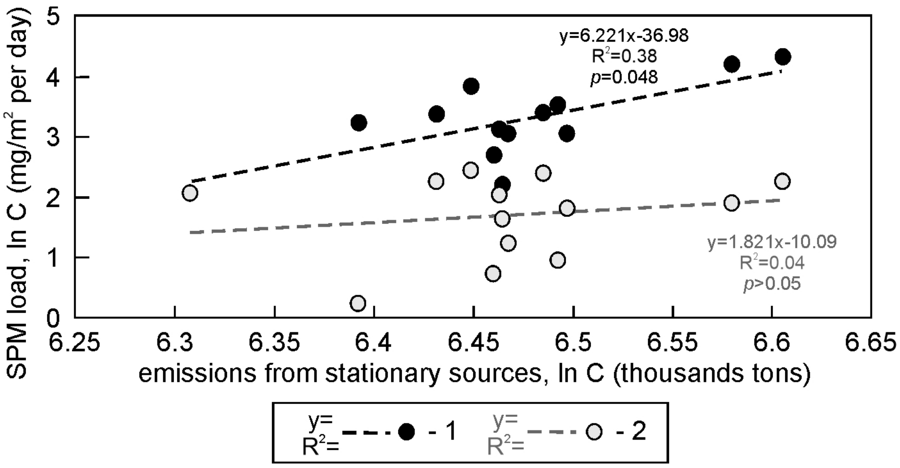

Figure S6. Relationship between the ln-transformed levels of suspended particulate matter (SPM) load at the urban (1) and suburban (2) stations and the ln-transformed total amount of emissions from stationary sources in Irkutsk Region.

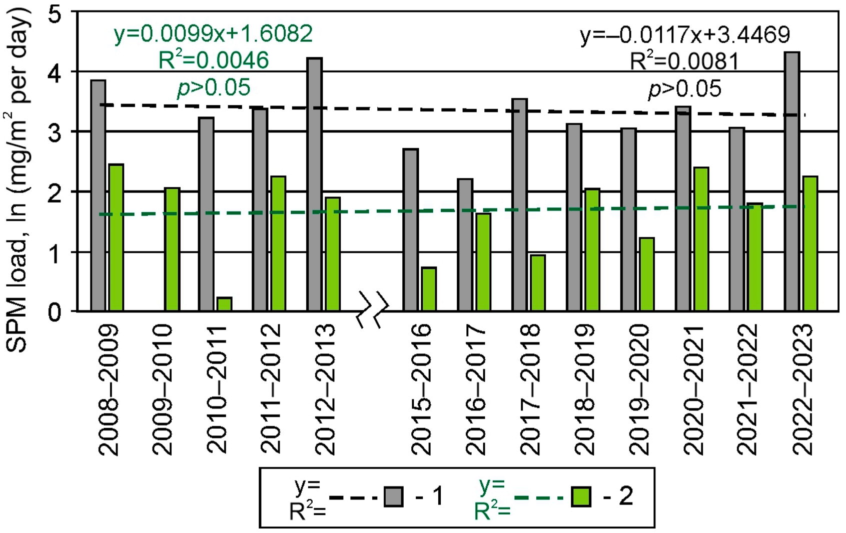

Figure S7. The load of suspended particulate matter (SPM) at the urban (1) and suburban (2) stations in winter in 2008–2023.

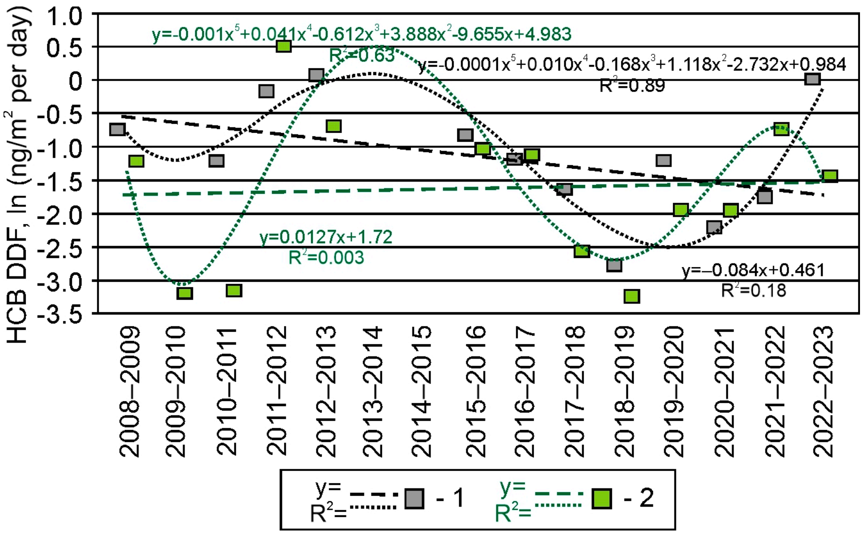

Figure S8. Temporal variations of DDFs of HCB in snow at the urban (1) and suburban (2) stations in 2009–2023.

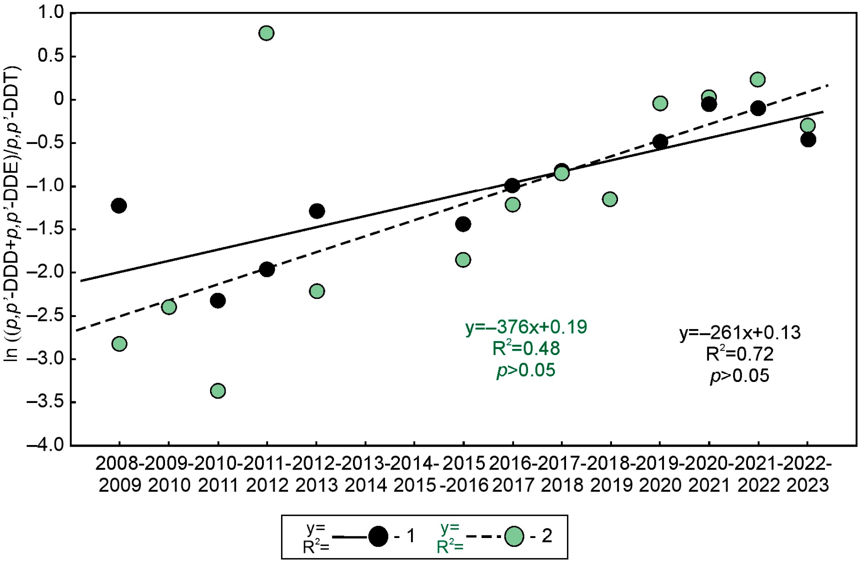

Figure S9. Temporal trend of the ratios of DDT and its metabolites in snow at the urban (1) and suburban (2) stations in 2009–2023.

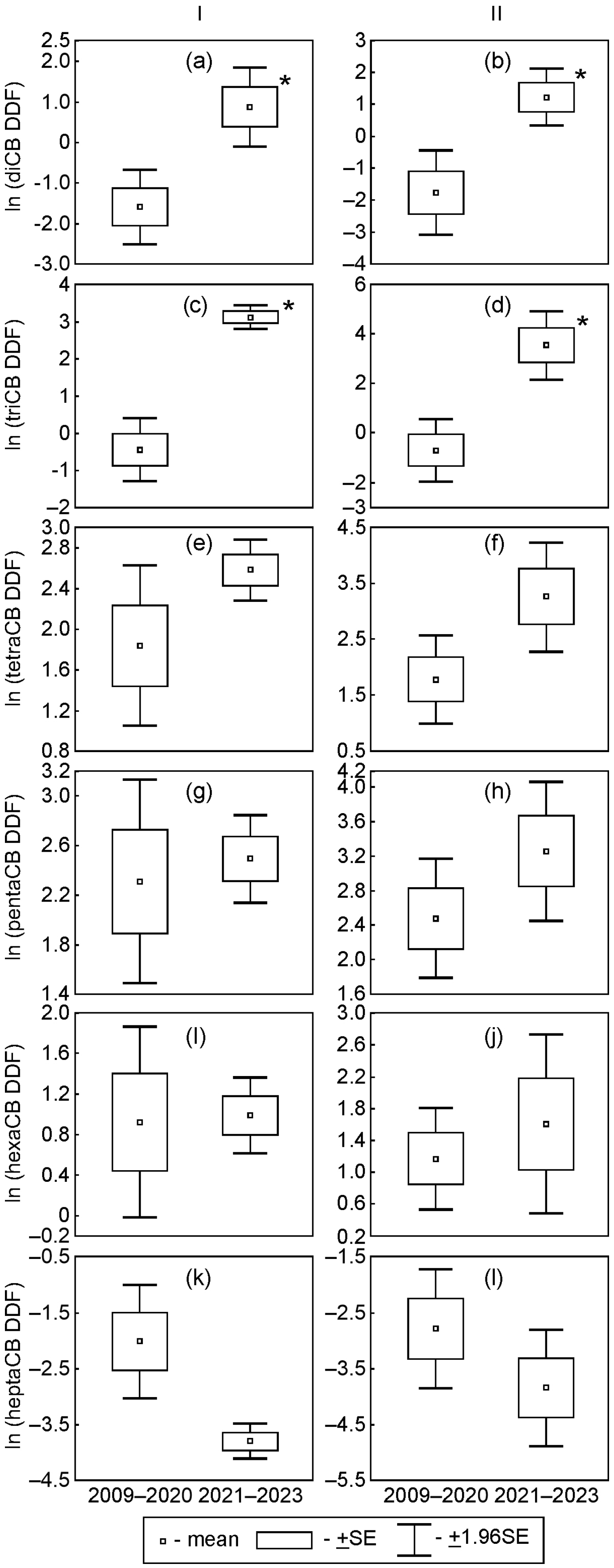

Figure S10. The comparison of mean values of daily deposition fluxes (DDF) of PCB homologues in snow at the urban (I) and suburban (II) stations in periods with different operational activities at the Usol'ekhimprom industrial area (Ln (ng/m^2 per day); *—p<0.05).

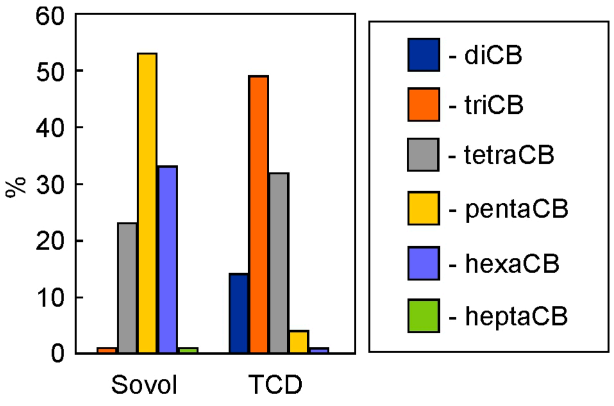

Figure S11. The relative PCB homological compositions in PCB technical mixtures of Sovol and Trichlorodiphenyl (TCD) [101].

Table S1. The list of PCB congeners and their groups and OCPs analyzed in snow samples, instrumental methods, and types of snow sampling companies (compounds highlighted in red were detected below MDLs at both stations in the given year)

Winter	Individual PCB congeners and its groups	OCPs	Instrumental method	Types of snow sampling companies
2008–2009	31 individual congeners and groups of PCBs: 8, 31, 28, 52, 49, 44, 37, 74, 70/76, 95/66, 101/90, 99, 97, 87/115, 85, 110/77, 82, 118, 153, 105, 179, 138, 158, 187, 183, 128, 156, 180, 169, 170, 189	HCB, *p,p*`-DDT, *p,p*`-DDD, *p,p*`-DDE, *α*-, *γ*-HCH	GC/μECD	[38]
2009–2010	31 individual congeners and groups of PCBs: 8, 31, 28, 52, 49, 44, 37, 74, 70/76, 95/66, 101/90, 99, 97, 87/115, 85, 110/77, 82, 118, 153, 105, 179, 138, 158, 187, 183, 128, 156, 180, 169, 170, 189	HCB, *p,p*`-DDT, *p,p*`-DDD, *p,p*`-DDE, *α*-, *γ*-HCH	GC/ECD	[38]
2010–2011	31 individual congeners and groups of PCBs: 8, 31, 28, 52, 49, 44, 37, 74, 70/76, 95/66, 101/90, 99, 97, 87/115, 85, 110/77, 82, 118, 153, 105, 179, 138, 158, 187, 183, 128, 156, 180, 169, 170, 189	HCB, *p,p*`-DDT, *p,p*`-DDD, *p,p*`-DDE, *α*-, *γ*-HCH	GC/ECD	[38]
2011–2012	37 individual congeners and groups of PCBs: 8, 31, 28, 52, 49, 47, 44, 37, 74, 70/76, 95/66, 101/90, 99, 97, 87/115, 85, 110/77, 82, 149, 118, 153, 132, 105, 141, 179, 138, 158, 187, 183, 128, 156, 180, 169, 170, 196, 189	HCB, *p,p*`-DDT, *p,p*`-DDD, *p,p*`-DDE, *α*-, *γ*-HCH	GC/ECD	This study
2012–2013	37 individual congeners and groups of PCBs: 8, 31, 28, 52, 49, 47, 44, 37, 74, 70/76, 95/66, 101/90, 99, 97, 87/115, 85, 110/77, 82, 149, 118, 153, 132, 105, 141, 179, 138, 158, 187, 183, 128, 156, 180, 169, 170, 196, 189	HCB, *p,p*`-DDT, *p,p*`-DDD, *p,p*`-DDE, *α*-, *γ*-HCH	GC/ECD	This study
2015–2016	37 individual congeners and groups of PCBs: 8, 31, 28, 52, 49, 47, 44, 37, 74, 70/76, 95/66, 91, 101/90, 99, 97, 87/115, 85, 110/77,	HCB, *p,p*`-DDT, *p,p*`-DDD, *p,p*`-DDE, *α*-, *γ*-HCH	GC/ECD	[37]

	82, 149, 118, 153, 132, 105, 141, 179, 138, 158, 187, 183, 128, 156, 180, 169, 170, 196, 189			
2016–2017	35 individual congeners and groups of PCBs: 8, 31, 28, 52, 49, 47, 44, 37, 74, 70/76, 95/66, 91, 101/90, 99, 97, 87/115, 85, 110/77, 82, 149, 118, 153, 132, 105, 141, 138, 158, 187, 183, 128, 156, 180, 169, 170, 189	HCB, *p,p*`-DDT, *p,p*`-DDD, *p,p*`-DDE, α-, γ-HCH	GC/ECD	This study
2017–2018	36 congeners of PCBs: 8, 11, 31, 28, 52, 49, 47, 44, 74, 66, 91, 56, 101, 99, 97, 87, 85, 110, 77, 149, 118, 153, 132, 105, 141, 138, 126, 187, 183, 128, 177, 156, 180, 169, 170, 196	HCB, *p,p*`-DDT, *o,p*`-DDT, *p,p*`-DDD, *o,p*`-DDD, *p,p*`-DDE, *o,p*`-DDE, α-, γ- HCH	GC/MC	This study
2018–2019	36 congeners of PCBs: 8, 11, 31, 28, 52, 49, 47, 44, 74, 66, 91, 56, 101, 99, 97, 87, 85, 110, 77, 149, 118, 153, 132, 105, 141, 138, 126, 187, 183, 128, 177, 156, 180, 169, 170, 196	HCB, *p,p*`-DDT, *o,p*`-DDT, *p,p*`-DDD, *o,p*`-DDD, *p,p*`-DDE, *o,p*`-DDE, α-, γ- HCH	GC/MC	This study
2019–2020	36 congeners of PCBs: 8, 11, 31, 28, 52, 49, 47, 44, 74, 66, 91, 56, 101, 99, 97, 87, 85, 110, 77, 149, 118, 153, 132, 105, 141, 138, 126, 187, 183, 128, 177, 156, 180, 169, 170, 196	HCB, *p,p*`-DDT, *o,p*`-DDT, *p,p*`-DDD, *o,p*`-DDD, *p,p*`-DDE, *o,p*`-DDE, α-, γ- HCH	GC/MC	This study
2020–2021	36 congeners of PCBs: 8, 11, 31, 28, 52, 49, 47, 44, 74, 66, 91, 56, 101, 99, 97, 87, 85, 77, 110, 149, 118, 153, 132, 105, 141, 138, 126, 187, 183, 128, 177, 156, 180, 169, 170, 196	HCB, *p,p*`-DDT, *o,p*`-DDT, *p,p*`-DDD, *o,p*`-DDD, *p,p*`-DDE, *o,p*`-DDE, α-, γ- HCH	GC/MC	[38]
2021–2022	37 congeners of PCBs: 8, 11, 31, 28, 52, 49, 47, 44, 37, 74, 70, 66, 91, 101, 99, 97, 87, 85, 77, 110, 82, 149, 118, 114, 153, 132, 105, 141, 138, 158, 126, 187, 183, 128, 177, 156, 180, 169, 170, 196, 189	HCB, *p,p*`-DDT, *o,p*`-DDT, *p,p*`-DDD, *o,p*`-DDD, *p,p*`-DDE, *o,p*`-DDE, α-, γ- HCH	GC/MC	This study
2022–2023	37 congeners of PCBs: 8, 11, 31, 28, 52, 49, 47, 44, 37, 74, 70, 66, 91, 101, 99, 97, 87, 85, 77, 110, 82, 149, 118, 114, 153, 132, 105, 141, 138, 158, 126, 187, 183, 128, 177, 156, 180, 169, 170, 196, 189	HCB, *p,p*`-DDT, *o,p*`-DDT, *p,p*`-DDD, *o,p*`-DDD, *p,p*`-DDE, *o,p*`-DDE, α-, γ- HCH	GC/MC	This study

Table S2. The significance of differences (t-test) and coefficients of correlation and the significance of differences and correlations between values obtained at the urban and suburban stations ("-"— p>0.05)

.

**Components**	**Comparison between values in urban and suburban, t-test, p**	**Relationship of values in urban and suburban stations**	
		R	p
height of snow cover	*	0.80	0.002
snow cover density	-	0.85	<0.001
SWE	-	0.92	<0.001
SPM	***	-	-
HCB	-	-	-
α-HCH	-	0.90	<0.001
γ-HCH	-	0.98	<0.001

α+γ-hch	-	0.96	<0.001
p,p′-DDT	-		
p,p′-DDE	-	0.78	0.003
p,p′-DDD	-	-	-
∑ *p,p′*-DDX	-	0.60	0.04
PCB-8 or 8+5	-	-	-
PCB-11	-	0.91	0.011
PCB-31	-	0.82	0.002
PCB-28	-	0.86	<0.001
PCB-52	-	0.83	0.001
PCB-49	-	0.85	<0.001
PCB-47	-	0.79	0.007
PCB-44	-	0.80	0.002
PCB-37	-	0.85	0.016
PCB-74	-	0.89	<0.001
PCB-70 or 70+76	-	0.84	0.019
PCB-66 or 95+66	-	0.84	0.001
PCB-91	-	0.95	<0.001
PCB-60	-	-	-
PCB-56	-	0.99	0.012
PCB-101 or 101+90	-	0.80	0.002
PCB-99	-	0.87	<0.001
PCB-97	-	-	-
PCB-87 or 87+115	-	0.84	0.001
PCB-85	-	0.85	<0.001
PCB-77	-	-	-
PCB-110 or 110+77	-	0.82	0.001
PCB-82	-	-	-
PCB-149	-	0.82	0.004
PCB-118	-	0.83	<0.001
PCB-114		-	-
PCB-153	-	0.78	0.003
PCB-132	-	0.84	0.003
PCB-105	-	0.80	0.002
PCB-141	-	0.69	0.026
PCB-179		-	-
PCB-138	-	0.61	0.035
PCB-158	-	-	-
PCB-126	-	-	-
PCB-187	-	-	-
PCB-183	-	0.65	0.042
PCB-128	-	0.74	0.009
PCB-177	-	-	-
PCB-156	-	0.68	0.020
PCB-180	-	0.68	0.015
PCB-169	-	-	-

PCB-170	-	-	-
PCB-196	-	-	-
PCB-189	-	-	-
PCB-194	-	-	-
∑ PCB	-	0.81	0.001
∑PCB_6	-	0.79	0.002
2CL	-	0.73	0.025
3CL	-	0.84	<0.001
4CL	-	0.87	<0.001
5CL	-	0.85	<0.001
6CL	-	0.76	0.004
7CL	-	0.66	0.019

Table S3. The mean, median, range (min-max), standard deviation (SD), and standard error (SE) values of levels of PCB congeners in snow found in 2009–2023 at the urban station (ng/L) (BDL—below detected level).

Compound	N	Mean	Median	Min	Max	SD	SE
PCB-8/5	9	1.72	0.68	0.07	9.03	2.83	0.94
PCB-11	6	0.74	0.78	0.05	1.49	0.66	0.27
PCB-31	11	5.13	1.88	BDL	20	7.27	2.19
PCB-28	12	7.24	1.51	0.10	29	11.3	3.25
PCB-52	12	6.53	6.03	0.94	13.4	4.32	1.25
PCB-49	12	2.12	2.16	0.36	3.89	1.43	0.41
PCB-47	10	0.62	0.71	0.07	1.30	0.38	0.12
PCB-44	12	3.68	3.64	0.37	9.06	2.58	0.74
PCB-37	7	1.12	0.70	BDL	3.74	1.29	0.49
PCB-74	12	2.58	2.03	0.14	6.90	2.15	0.62
PCB-70 or 70+76	7	3.34	3.11	BDL	8.75	2.84	1.07
PCB-66 or 95+66	12	3.11	2.50	BDL	10.8	3.44	0.99
PCB-91	8	0.81	0.42	BDL	2.45	0.89	0.32
PCB-60	5	1.47	1.22	0.56	2.79	0.88	0.39
PCB-56	4	0.17	0.13	0.04	0.37	0.15	0.08
PCB-101 or 101+90	12	7.02	4.13	1.21	22	6.27	1.81
PCB-99	12	5.58	3.33	0.78	18	5.28	1.52
PCB-97	12	1.76	0.99	0.15	6.17	1.83	0.53
PCB-87 or 87+115	12	1.54	0.98	0.04	5.52	1.65	0.48
PCB-85	12	1.39	0.80	0.08	5.32	1.55	0.45
PCB-77	6	0.04	0.05	BDL	0.10	0.04	0.01
PCB-110 or 110+77	12	7.10	3.53	1.25	22	7.15	2.06
PCB-82	7	0.17	0.14	BDL	0.44	0.15	0.06
PCB-149	10	1.98	1.18	0.38	6.23	1.94	0.61
PCB-118	12	6.13	3.19	0.54	24	7.06	2.04

PCB-114	3	0.16	0.22	BDL	0.27	0.14	0.08
PCB-153	12	2.74	1.31	0.35	11	3.24	0.93
PCB-132	10	0.54	0.33	0.04	2.82	0.82	0.26
PCB-105	12	2.17	1.04	0.04	8.95	2.65	0.76
PCB-141	10	0.35	0.12	BDL	1.22	0.47	0.15
PCB-179	2	0.02	0.02	0.01	0.02	0.01	0.004
PCB-138	12	3.56	1.56	0.11	16	4.57	1.32
PCB-158	7	0.22	0.08	BDL	0.89	0.31	0.12
PCB-126	7	BDL	BDL	BDL	BDL	-	-
PCB-187	10	0.10	0.02	BDL	0.40	0.14	0.05
PCB-183	10	0.09	0.03	BDL	0.29	0.12	0.04
PCB-128	11	0.58	0.21	BDL	2.51	0.76	0.23
PCB-177	6	BDL	BDL	BDL	BDL	-	-
PCB-156	11	0.25	0.13	BDL	1.07	0.38	0.12
PCB-180	12	0.26	0.07	BDL	1.26	0.40	0.12
PCB-169	8	BDL	BDL	BDL	BDL	-	-
PCB-170	11	0.14	0.02	BDL	0.65	0.23	0.01
PCB-196	8	BDL	BDL	BDL	BDL	-	-
PCB-189	4	BDL	BDL	BDL	BDL	-	-
PCB-194	7	BDL	BDL	BDL	BDL	-	-

Table S4. The mean, median, range (min-max), standard deviation (SD), and standard error (SE) values of levels of PCB congeners in snow found in 2009–2023 at the suburban station (ng/L) (BDL —below detected level).

Compound	N	Mean	Median	Min	Max	SD	SE
PCB-8 or 8+5	9	2.68	0.42	BDL	17	5.39	1.80
PCB-11	6	0.81	0.51	BDL	2.23	0.90	0.37
PCB-31	12	13	1.16	BDL	120	34	9.8
PCB-28	13	17	1.15	0.08	183	50	14
PCB-52	13	8.87	5.86	0.78	39	11	2.96
PCB-49	13	3.48	1.99	0.19	20	5.28	1.46
PCB-47	10	1.07	0.59	0.08	6.3	1.85	0.59
PCB-44	13	4.94	2.81	0.60	27	7.09	1.97
PCB-37	7	2.94	0.96	0.03	15	5.37	2.03
PCB-74	13	3.54	2.68	0.15	13	3.93	1.09
PCB-70 or 70+76	8	4.93	2.01	BDL	26	8.61	3.04
PCB-66 or 95+66	12	4.25	2.31	BDL	15	4.95	1.43
PCB-91	8	1.05	0.56	BDL	2.95	1.07	0.38
PCB-60	5	2.12	0.84	0.71	5.94	2.24	1.00
PCB-56	4	0.19	0.12	0.03	0.49	0.22	0.11
PCB-101 or 101+90	13	8.02	5.90	0.45	26	8.32	2.31
PCB-99	13	6.53	4.66	0.65	21	6.65	1.84
PCB-97	13	1.86	1.49	BDL	5.85	1.88	0.52

PCB-87 or 87+115	13	1.91	0.81	BDL	9.66	2.60	0.72
PCB-85	12	1.84	1.38	0.11	6.02	2.00	0.58
PCB-77	6	0.14	0.004	BDL	0.72	0.29	0.12
PCB-110 or 110+77	13	8.36	6.01	1.05	23	7.39	2.05
PCB-82	7	0.36	0.26	BDL	1.12	0.36	0.14
PCB-149	10	1.97	1.39	0.55	4.85	1.59	0.50
PCB-118	13	8.40	4.67	1.16	28	9.03	2.51
PCB-114	3	0.58	0.35	BDL	1.39	0.72	0.42
PCB-153	13	2.92	1.81	0.71	9.16	2.78	0.77
PCB-132	10	0.61	0.27	0.08	2.14	0.78	0.24
PCB-105	13	2.89	1.64	0.44	9.58	2.97	0.82
PCB-141	10	0.31	0.25	BDL	0.82	0.31	0.10
PCB-179	3	0.07	0.09	0.03	0.09	0.04	0.02
PCB-138	13	3.84	2.51	0.90	13	3.76	1.04
PCB-158	8	0.29	0.20	BDL	1.01	0.33	0.12
PCB-126	8	BDL	BDL	BDL	BDL	-	-
PCB-187	11	0.03	0.002	BDL	0.16	0.06	0.02
PCB-183	11	0.04	0.005	BDL	0.23	0.07	0.02
PCB-128	11	0.62	0.45	BDL	1.54	0.54	0.16
PCB-177	6	BDL	BDL	BDL	BDL	-	-
PCB-156	11	0.25	0.14	BDL	0.58	0.24	0.07
PCB-180	13	0.12	0.05	BDL	0.61	0.17	0.05
PCB-169	10	0.03	0.002	BDL	0.19	0.06	0.02
PCB-170	12	0.04	0.006	BDL	0.22	0.07	0.02
PCB-196	9	0.20	0.008	BDL	0.75	0.31	0.10
PCB-189	4	BDL	BDL	BDL	BDL	-	-
PCB-194	8	BDL	BDL	BDL	BDL	-	-

Table S5. The significant (p<0.05) correlation coefficients of DDFs of POPs in snow at the urban station ('-"—p>0.05)

	**SPM**	**HCB**	**α+γ-HCH**	***p,p′*-DDE**	***p,p′*-DDT**	**∑*p,p′*-DDX**	**PCB-11**	**∑PCB$_{all}$**	**∑PCB$_6$**	**diCB**	**triCB**	**tetraCB**	**pentaCB**	**hexaCB**	**heptaCB**
SPM		-	-	-	-	-	-	-	-	-	-	-	-	-	-
HCB	-		0.70	-	0.70	0.68	-	-	-	-	-	-	-	-	-
α+γ-hch	-	0.70		-	0.71	0.75	-	-	-	-	-	-	-	-	0.59
p,p′-DDE	-	-	-		0.77	0.89	0.81	0.61	0.62	-	-	0.64	0.67	-	0.61
p,p′-DDT	-	0.70	0.71	0.77		0.92	-	0.60	0.59	-	-	0.76	0.66	-	-
∑*p,p′*-DDX	-	0.68	0.75	0.89	0.92		-	-	-	-	-	0.66	0.65	-	0.64
PCB-11	-	-	-	0.81	-	-		0.91	0.92	-	0.81	0.94	-	-	-
∑PCBs	-	-	-	0.61	0.60	-	0.91		0.99	-	0.80	0.94	0.94	0.88	-
∑PCB$_6$	-	-	-	0.62	0.59	-	0.92	0.99		-	0.84	0.92	0.89	0.83	-
diCB	-	-	-	-	-	-	-	-	-		0.86	-	-	-	-
triCB	-	-	-	-	-	-	0.81	0.80	0.84	0.86		0.70	-	-	-
tetraCB	-	-	-	0.64	0.76	0.66	0.94	0.94	0.92	-	0.70		0.89	0.78	-
pentaCB	-	-	-	0.67	0.66	0.65	-	0.94	0.89	-	-	0.89		0.96	0.72
hexaCB	-	-	-	-	-	-	-	0.88	0.83	-	-	0.78	0.96		0.75
heptaCB	-	-	0.59	0.61	-	0.64	-	-	-	-	-	-	0.72	0.75	

Table S6. The significant (p<0.05) correlation coefficients of DDFs of POPs in snow at the suburban station ('-" — p>0.05)

	**SPM**	**HCB**	**$\alpha+\gamma$-hch**	***p,p'*-DDE**	***p,p'*-DDT**	**$\sum$*p,p′*-DDX**	**PCB-11**	**$\sum PCB_{all}$**	**$\sum PCB_6$**	**diCB**	**triCB**	**tetraCB**	**pentaCB**	**hexaCB**	**heptaCB**
SPM		-	-	-	-	-	-	-	-	-	-	-	-	-	-
HCB	-		-	0.59	-	-	0.87	0.82	0.78	-	0.70	0.83	0.86	0.81	0.73
$\alpha+\gamma$-hch	-	-		-	0.66	0.66	-	-	-	-	-	-	-	-	0.76
p,p′-DDE	-	0.59	-		-	0.71	0.84	0.65	0.67	0.74	0.62	0.62	0.63	0.62	-
p,p'-DDT	-	-	0.66	-		0.97	-	-	-	-	-	-	-	-	-
$\sum$*p,p′*-DDX	-	-	0.66	0.71	0.97		-	-	-	-	-	-	-	-	-
PCB 11	-	0.87	-	0.84	-	-		-	-	0.86	0.82	0.88	-	-	-
$\sum$PCBs	-	0.82	-	0.65	-	-	-		0.99	0.88	0.89	0.98	0.97	0.88	-
$\sum PCB_6$	-	0.78	-	0.67	-	-	-	0.99		0.92	0.92	0.96	0.95	0.87	-
diCB	-	-	-	0.74	-	-	0.86	0.88	0.92		0.95	0.86	0.75	-	-
triCB	-	0.70	-	0.62	-	-	0.82	0.89	0.92	0.95		0.87	0.79	0.67	-
tetraCB	-	0.83	-	0.62	-	-	0.87	0.98	0.96	0.86	0.87		0.93	0.81	-
pentaCB	-	0.86	-	0.63	-	-	-	0.97	0.95	0.75	0.79	0.93		0.95	-
hexaCB	-	0.81	-	0.62	-	-	-	0.88	0.87	-	0.67	0.81	0.95		
heptaCB	-	0.73	0.76	-	-	-	-	-	-	-	-	-	-	-	

Table S7. Results of linear regression analysis comparing ln-transformed DDFs of POP values and years of investigation in 2009–2023 (* $p < 0.05$, ** $p < 0.01$, *** $p < 0.001$, «-» $p > 0.05$).

**Compounds**	**b, slope**	**p-value**	**R^2**
		**Urban station**	
α-HCH	–0.513	***	0.77
γ-HCH	–0.496	**	0.59
$\alpha+\gamma$-HCH	–0.489	***	0.71
p,p′-DDD	–0.317	*	0.45
(DDE+DDD)/DDT	0.129	**	0.72
PCB-28	0.278	*	0.43
PCB-180	–0.249	*	0.34
triCB	0.279	*	0.42
		**Suburban station**	
α-HCH	–0.447	***	0.67
γ-HCH	–0.438	**	0.52
$\alpha+\gamma$-HCH	–0.430	**	0.63
p,p′-DDT	–0.189	*	0.44
$\sum$*p,p*\`-DDX	–0.154	*	0.34
(DDE+DDD)/DDT	0.186	**	0.48
PCB-28	0.346	*	0.40
PCB-70+76	0.231	*	0.58
PCB-180	–0.297	*	0.43
PCB-170	–0.335	*	0.42
triCB	0.335	*	0.38

Table S8. The list of the first ten most abundant PCB congeners and their groups in snow from max to min contribution to total PCB levels in 2009–2023 at the urban (U) and suburban (S) stations (diCB highlighted in orange, triCB highlighted in yellow, tetraCB highlighted in green, pentaCB highlighted in blue, hexaCB highlighted in red; U—urban, S—suburban)

**Station**	**U**	**S**	**S**	**U**	**S**	**U**	**S**	**U**	**S**
Winter	2008–2009	2008–2009	2009–2010	2010–2011	2010–2011	2011–2012	2011–2012	2012–2013	2012–2013
Congeners	52	110/77	118	101/90	101/90	110/77	110/77	110/77	110/77
	101/90	118	110/77	95/66	95/66	101/90	52	52	101/90
	44	101/90	138	52	52	118	118	101/90	118
	110/77	138	153	110/77	110/77	52	101/90	99	99
	95/66	52	52	118	118	99	95/66	95/66	52
	99	153	70/76	99	99	95/66	99	118	95/66
	118	99	99	138	44	138	44	44	138
	49	95/66	44	44	138	44	138	138	153
	153	44	101/90	153	87/115	153	105	153	105
	28	105	105	87/115	153	105	74	74	74
Sum of % of first 10 PCB congeners	82%	77%	81%	79%	74%	63%	54%	59%	61%
% of PCB-28 + PCB-31	2.4	4.4	0.9	0.5	0.6	2.5	3.3	4,8	2.1%

Table S8. Continued.

**Station**	**U**	**S**	**U**	**S**	**U**	**S**	**U**	**S**	**U**	**S**
Winter	2016–2017	2016–2017	2015–2016	2015–2016	2016–2017	2016–2017	**2017–2018**	2017–2018	**2018–2019**	2018–2019
Congeners	52	95/66	110/77	101/90	52	95/66	**101/90**	118	**110**	118
	110/77	52	101/90	110/77	110/77	52	**110**	110	**118**	101
	101/90	28	52	118	101/90	28	**118**	101	**101**	110
	95/66	110/77	99	99	95/66	110/77	**99**	99	**99**	99
	99	101/90	95/66	52	99	101/90	**52**	138	**52**	52
	44	31	118	95/66	44	31	**138**	153	**138**	138
	118	99	44	138	118	99	**153**	52	**153**	153
	74	44	74	74	74	44	**74**	105	**105**	105
	49	118	87/115	44	49	118	**105**	149	**49**	74
	138	74	70/76	105	138	74	**44**	74	**97**	149
Sum of % of first 10 PCB congeners	60%	74%	62%	60%	60%	74%	**61%**	78%	**62%**	69%
% of PCB-28 + PCB-31	4	15	1.9	1.1	4	15	**4**	1.9	**4.8**	1.3

Table S8. Continued.

**Station**	**U**	**S**	**U**	**S**	**U**	**S**	**U**	**S**
Winter	**2019–2020**	2019–2020	**2020–2021**	2020–2021	**2021–2022**	2021–2022	**2022–2023**	2022–2023
Congeners	**118**	118	**28**	28	**28**	28	**28**	28
	**101**	101	**31**	31	**31**	31	**31**	31
	**110**	110	**8**	52	**52**	52	**52**	52
	**99**	99	**52**	101	**101**	118	**101**	101
	**138**	52	**101**	110	**70**	44	**70**	70
	**52**	138	**44**	118	**118**	101	**44**	118
	**153**	74	**99**	99	**110**	70	**110**	110
	**105**	153	**110**	44	**99**	110	**118**	99
	**74**	105	**49**	49	**44**	99	**99**	44
	**149**	44	**118**	74	**66**	49	**49**	66
Sum of % of first 10 PCB congeners	**66%**	62%	**67%**	71%	**62%**	52%	**53%**	53%
% of PCB-28 + PCB-31	**2.4**	3.6	**52%**	29%	**31%**	46%	**40%**	32%

Table S9. Results of the multiple linear regression analysis comparing the average temperature of air, ln-transformed total values of precipitation, ln-transformed average air humidity, and ln-transformed total sunshine duration for 4 months (November + December + January + February) during the periods of study in 2008–2023 and ln-transformed values of POP DDFs (* $p < 0.05$, ** $p < 0.01$, *** $p < 0.001$, "-", $p > 0.05$).

**Compound**		**slope, b**	***p*-value**	**R^2**	**slope, b**	***p*-value**	**R^2**
			suburban			urban	
HCB	Precipitation volume				–0.29	-	0.88
	Air humidity				14.5	**	
	Sunshine duration				0.07	-	
	Temperature				–0.12	-	
α-HCH	Precipitation volume	–3.83	-	0.79	–4.19	-	0.90
	Air humidity	48.6	**		47.7	**	
	Sunshine duration	–1.07	-		–6.14	-	
	Temperature	–0.09	-		–0.54	-	
γ-HCH	Precipitation volume	–4.74	-	0.70	–5.96	-	0.75
	Air humidity	54.0	**		54.9	**	
	Sunshine duration	–4.54	-		–8.10	-	
	Temperature	0.10	-		–0.11	-	
α+γ-HCH	Precipitation volume	–3.62	-	0.77	–4.63	-	0.83
	Air humidity	48.8	**		48.5	**	
	Sunshine duration	–1.51	-		–5.98	-	
	Temperature	0,019	-		–0.31	-	
p,p′-DDT	Precipitation volume	–6.09	*	0.75	–4.01	-	0.81
	Air humidity	27.2	**		47.6	**	
	Sunshine duration	–7.94	-		–12.8	-	
	Temperature	0.15	-		0.35	-	
Total *p,p′*-DDX	Precipitation volume	–6.23	**	0.79	–3.40	-	0.70
	Air humidity	26.8	**		26.2	*	

	Sunshine duration	–9.07	*		–7.17	-	
	Temperature	0.24	-		–0.01	-	
PCB-8	Precipitation volume	–9.69	*	0.92			
	Air humidity	36.7	*				
	Sunshine duration	–26.2	*				
	Temperature	1.00	*				
PCB-52	Precipitation volume	–2.20	-	0.55			
	Air humidity	11.7	-				
	Sunshine duration	–6.09	-				
	Temperature	0.59	*				
PCB-49	Precipitation volume	–1.80	-	0.46			
	Air humidity	8.53	-				
	Sunshine duration	–5.53	-				
	Temperature	0,63	*				
PCB-47	Precipitation volume	–7.29	***	0.98	–5.60	*	0.92
	Air humidity	31.0	***		23.6	**	
	Sunshine duration	–15.6	***		–14.4	**	
	Temperature	0.73	***		0.25	-	
PCB-44	Precipitation volume	–2.28	-	0.54			
	Air humidity	12.5	-				
	Sunshine duration	–6.58	-				
	Temperature	0.60	*				
PCB-66	Precipitation volume	–9.79	-	0.62	–0.94	-	0.73
	Air humidity	61.2	*		46.4	*	
	Sunshine duration	–6.65	-		2.39	-	
	Temperature	0.44	-		–0.03	-	
PCB-101	Precipitation volume	–0.29	-	0.50			
	Air humidity	6.52	-				
	Sunshine duration	–2.38	-				
	Temperature	0.55	*				
PCB-87	Precipitation volume	–4.29	-	0.78	–2.17	-	0.57
	Air humidity	60.3	**		27.8	*	
	Sunshine duration	–15.9	-		–4.85	-	
	Temperature	1.06	*		0.25	-	
PCB-110	Precipitation volume	–0.73	-	0.53			
	Air humidity	11.1	-				
	Sunshine duration	–2.76	-				
	Temperature	0.48	*				
PCB-149	Precipitation volume	–3.45	-	0.81			
	Air humidity	17.6	*				
	Sunshine duration	–8.76	*				
	Temperature	0.34	-				
PCB-118	Precipitation volume	–0.77	-	0.48			
	Air humidity	8.25	-				
	Sunshine duration	–3.12	-				
	Temperature	0.46	*				
PCB-169	Precipitation volume	–6.00	*	0.95	1.08	-	0.82
	Air humidity	40.5	**		–4.36	-	
	Sunshine duration	–12.6	*		–0.34	-	
	Temperature	–0.53	*		0.27	*	
PCB-170	Precipitation volume	–5.21	-	0.70			

	Air humidity	45.8	*	
	Sunshine duration	–10.9	-	
	Temperature	0.19	-	
Total PCB_{all}	Precipitation volume	–1.49	-	0.53
	Air humidity	10.1	-	
	Sunshine duration	–4.80	-	
	Temperature	0.59	*	
Total PCB_6	Precipitation volume	–1.86	-	0.56
	Air humidity	9.65	-	
	Sunshine duration	–5.80	-	
	Temperature	0.61	*	
diCB	Precipitation volume	–9.02	*	0.93
	Air humidity	28.6	*	
	Sunshine duration	–22.6	*	
	Temperature	0.99	*	
triCB	Precipitation volume	–2.53	-	0.48
	Air humidity	9.19	-	
	Sunshine duration	–7.91	-	
	Temperature	1.12	*	
tetraCB	Precipitation volume	–2.24	-	0.52
	Air humidity	14.0	-	
	Sunshine duration	–5.85	-	
	Temperature	0.62	*	
pentaCB	Precipitation volume	–0.61	-	0.49
	Air humidity	8.66	-	
	Sunshine duration	–2.74	-	
	Temperature	0.48	*	

Table S10. Results of the multiple linear regression analysis comparing the ln-transformed values of the precipitation volumes for November and December of previous year and January and February of study year in 2008–2023 and ln-transformed values of POP DDFs (* $p < 0.05$, ** $p < 0.01$, "-", $p > 0.05$).

**Compound**		**slope, b**	***p*-value**	**R^2**	**slope, b**	***p*-value**	**R^2**
		suburban			urban		
HCB	November	–0.19	-	0.45			
	December	–0.68	-				
	January	–1.35	-				
	February	1.40	*				
p,p′-DDE	November	–0.99	-	0.53			
	December	–1.01	-				
	January	–1.41	-				
	February	1.23	*				
PCB-31	November	0.60	-	0.70			
	December	–3.01	-				
	January	–2.11	-				
	February	5.08	*				
PCB-28	November	–0.45	-	0.48			
	December	–2.64	-				
	January	–2.11	-				
	February	2.98	*				

PCB-52	November	–0.69	-	0.62
	December	–0.84	-	
	January	–1.25	-	
	February	1.62	*	
PCB-49	November	–0.83	-	0.57
	December	–1.15	-	
	January	–1.33	-	
	February	1.77	*	
PCB-44	November	–0.62	-	0.63
	December	–1.09	-	
	January	–1.04	-	
	February	1.64	*	
PCB-74	November	–0.54	-	0.56
	December	–0.79	-	
	January	–1.61	-	
	February	1.82	*	
PCB-101	November	–0.48	-	0.61
	December	–0.50	-	
	January	–1.56	-	
	February	1.67	*	
PCB-99	November	–0.60	-	0.62
	December	–0.42	-	
	January	–1.29	-	
	February	1.46	*	
PCB-87	November	1.18	-	0.53
	December	–0.55	-	
	January	–0.68	-	
	February	3.60	*	
PCB-85	November	–0.36	-	0.67
	December	–0.74	-	
	January	–1.86	*	
	February	1.87	**	
PCB-110	November	–0.54	-	0.71
	December	–0.41	-	
	January	–1.38	*	
	February	1.50	**	
PCB-118	November	–0.71	-	0.70
	December	–0.31	-	
	January	–1.29	*	
	February	1.37	**	
PCB-153	November	–0.69	-	0.63
	December	–0.22	-	
	January	–0.99	-	
	February	1.11	*	
PCB-105	November	–0.67	-	0.65
	December	–0.28	-	
	January	–1.16	-	
	February	1.39	*	
PCB-138	November	–0.73	-	0.60
	December	–0.18	-	
	January	–0.99	-	

	February	1.14	*	
PCB-156	November	–0.35	-	0.56
	December	–1.25	-	
	January	–1.89	-	
	February	2.93	*	
Total PCB_{all}	November	–0.59	-	0.58
	December	–0.72	-	
	January	–1.25	-	
	February	1.63	*	
Total PCB_6	November	–0.61	-	0.57
	December	–0.78	-	
	January	–1.21	-	
	February	1.62	*	
triCB	November	–0.50	-	0.52
	December	–2.52	-	
	January	–2.17	-	
	February	3.18	*	
tetraCB	November	–0.55	-	0.55
	December	–0.94	-	
	January	–1.16	-	
	February	1.66	*	
pentaCB	November	–0.54	-	0.66
	December	–0.41	-	
	January	–1.34	-	
	February	1.48	**	
hexaCB	November	–0.71	-	0.62
	December	–0.28	-	
	January	–1.12	-	
	February	1.25	*	

Text S1. Backward and Forecast Air Trajectories

The 48 h forecast air trajectories of the emissions from the land source in the Usol′ekhimprom industrial area situated 1 m above ground level were generated every 6 h in the day and the day before each snow precipitation event in the winter of 2020–2021 (Figure S1). The model indicated the air distribution of emissions from the Usol′ekhimprom industrial area over a considerable area of the southern part of the Irkutsk Region and reached elevations between 500 and 1000 m above ground level within one day. So, backward air trajectories for urban and suburban sampling areas were generated every 6 h during each snow event at elevations of 500, 800, 1000, and 1500 m above ground level on one of the days with the highest precipitation volumes in January of 2021 (Figure S2) and in locations situated 800 m above ground level on several days with the highest precipitation volumes in the winter of 2020–2021 (Figure S3). It was found that the northern, northwestern, and western directions were the predominant directions of air masses in winter in the urban and suburban stations studied, confirming the effect of the emissions from the Usol′ekhimprom industrial area on the southern part of the Irkutsk Region including the stations studied.

Text S2. Characteristics of snow cover at the time of sampling and some meteorological indexes in 2009-2023.

The means ± standard deviations of the height of snow cover, SWE, and density of snow amounted to 0.33 ± 0.05 m, 55 ± 13 mm, and 0.17 ± 0.02 g/cm^3, respectively at the urban station and 0.40 ± 0.10 m, 62 ± 22 mm, and 0.15 ± 0.03 g/cm^3, respectively at the suburban station (Table 1). There was no significant difference between the ln-transformed means of SWE and density of snow cover at the urban and suburban stations ($p > 0.05$). The height and SWE were usually slightly higher at the suburban station than at the urban station. And, on the contrary, the density of snow at the urban station was slightly higher than that at the suburban station.

There was interannual variation in meteorological indexes, especially in winter months during the study period of January 2009–February 2023. For example, the difference between the highest and lowest average temperatures in December and January made up to 12.2 and 10.3 °C, respectively (Figure S5a). The differences between the highest and the lowest average month precipitation volumes and durations of sunshine were up to 15 times in February and 1.83 times in January, respectively (Figure S5bc). The sums of precipitation volume for four months with stable snow cover before snow sampling (November and December of the previous year + January and February of the year of sampling) varied between 39.4 mm in the winter of 2018–2019 and 104.3 mm in the winter of 2017–2018 with a mean value of 67.5 mm [42]. The sum of the duration of sunshine for these four months ranged between 358 hours in the winter of 2020–2021 and 536 hours in the winter of 2020–2019 with a mean value of 449 hours [44].

The interannual variations in meteorological indexes result in considerable interannual variations in snow cover characteristics. For example, the lowest SWE (Figure S6), the height of snow cover, and the density of snow at the urban and the suburban stations observed in winter of 2018–2019 amounted to 26 and 21 mm, 23 and 21 cm, and 0.11 and 0.10 g/cm^3, respectively. In the winter of 2011–2012, the highest SWEs were 72 and 115 mm at the urban and suburban stations, respectively. The biggest snow cover heights at the urban and suburban stations were found in winter in 2017–2018 (42 cm) and 2011–2013 (115 cm). The snow density amounted to 0.20 g/cm^3 in the winter of 2020–2021 at the urban station and 0.19 g/cm^3 in the winter of 2008–2009 at the suburban station. We found no relationship between SWE, density, and height of snow cover and the number of days of stable snow cover before sampling at either the urban or suburban station ($p > 0.05$), but there was a significant correlation between these snow cover characteristics at the urban and suburban stations ($p < 0.05$). The number of days with stable snow cover before

sampling varied between 91 days in the winter of 2022–2023 (the date of the sampling was February 26, 2023) and 129 days in the winter of 2016–2017 (the date of sampling was February 27, 2017) (Figure S6).

Text S3. The set of organochlorines and the frequency determination.

HCB, *p,p*`-DDE, and 18 individual PCB congeners levels above the MDLs were found in every sample investigated. *p,p*`-DDT levels above the MDLs were determined in 96% of samples, followed by γ-HCH, *o,p*`-DDT, $\alpha$-HCH, and *p,p*`-DDD (60%, 58%, 52%, and 43%, respectively). *o,p*`-DDD and *o,p*`-DDE levels below the MDLs were found in every sample. The numbers of investigated PCB congeners varied from 31 congeners in 2009–2011 to 37 congeners in 2012–2013, 2016, and 2021–2022 (Table S1). The number of PCB congeners and their groups below the MDLs in samples from both urban and suburban stations also varied from 1–2 in 2012–2016 to 13–14 PCB congeners and their groups in 2009–2010 (highlighted in red in Table S1).

Text S4. The change in relative homological and congener PCB patterns in snow samples.

The PCB homological compositions at both stations were analyzed using the cluster method (Figure 3) with the inclusion of relative PCB compositions of two technical PCB mixtures (Sovol and trichlorodiphenyl (TCD)) used in the former USSR [101]. The snow samples at both stations collected in 2009–2023 formed two groups in accordance with the relative PCB homological compositions. The first group was characterized by the domination of pentaCBs (means = 49 and 53% of total PCBs at the urban and suburban stations, respectively) followed by tetraCBs (33 and 27%) and hexaCBs (13 and 15%), and included snow sampled in the Februarys of 2009–2020 (Figure 3). Similar PCB homological patterns were previously found in snow samples from Usol'e-Sibirskoe [22]. The first group contained, also, Sovol with the predominance of pentaCBs (Figure S11). The portions of tetraCBs and pentaCBs in snow samples differed from those in Sovol [101] and soil samples from the Usol'e-Sibirskoe area [22] due to the different physical–chemical properties of tetraCBs and hexaCBs (lower chlorinated congeners more easily evaporate from surfaces and are transferred by air than higher chlorinated congeners [86]) resulting in an increase in the tetraCB portion and decrease in the hexaCB portion in snow relative to the primary PCB technical mixture or soils polluted with Sovol in the past.

Snow samples collected at the end of winter in 2020–2021, 2021–2022, and 2022–2023 were included in the second group (Figure 3). In this group, triCBs (means = 42 and 37 % of total PCBs at the urban and suburban stations, respectively) dominated amongst PCB homologues, followed by tetraCBs (25 and 27%) and pentaCBs (22 and 27%) (Fig. 1).

The lists of the first ten PCB congeners and their groups, being the biggest contributors to total PCB concentration, are presented in Table S8. In 2008–2020, the list of the most abundant PCB congeners consisted of pentachlorinated congeners including PCB-101/90, 110, 99, and 118; tetrachlorinated congeners including PCB-44, 52, and 66; and hexachlorinated congeners including PCB-138 and 153. They contributed more than 5% each in total PCB concentration every, or about every, year. Trichlorinated congeners (PCB-28 and 31) were included in the list of the first ten congeners in the winter of 2016–2017 at the suburban station and in the winter of 2008–2009 at the urban station only. This period corresponded to low productivities and then the shutting down of production at Usol'ekhimprom and bankruptcy of the enterprise in 2012, the period of uncontrolled waste storage starting in 2013, and the introduction of municipal and regional states of emergency starting in 2018 due to the threat of chemical pollution [34].

Starting from the winter of 2020–2021, the snow samples were dominated by triCB congeners (PCB-28 and PCB-31) at both stations, as well as a diCB congener (PCB-8) in the winter of 2020–2021 at the urban station, followed by tetraCB (PCB-52) and pentaCBs

(PCB-101, 110, 118, 99) every year at both stations. In these years, PCB-138 and PCB-153 were not detected amongst the most abundant PCB congeners. In the winters of 2020–2023, the sum of PCB-28 and PCB-31 contributed up to 31–52% and 29–46% to the total PCB concentrations at the urban and suburban stations, respectively in comparison with 0.6–4.8% in previous winters, with the exception of the winter in 2016–2017, when the portion of PCB-28 + PCB-31 amounted to 15% of the total PCB concentration in snow at the suburban station (Table S8). The same changes in PCB congener composition were found in the majority of snow samples collected in the southern part of the Irkutsk Region in the winter of 2020–2021 [38]. We considered, then, that the increase in the emissions of lower chlorinated compounds in snow sampled in the southern part of the Irkutsk Region was a result of the application of water curtains during the dismantling of buildings in Usol'ekhimpron to avoid the aerial transport of particles containing mercury [34,38], but this technique was used only during the dismantling of the mercury electrolysis workshop building in the fall of 2020 [34].

References

37. Mamontova, E.A.; Tarasova, E.N.; Mamontov, A.A. Concentration of persistent organic pollutants in soil, snow water, and vegetation in southern Baikal region. *Meteorol. Hydrol.* **2019**, *2*, 86–98. (In Russian).
38. Mamontova, E.A.; Mamontov, A.A. Spatial and temporal variations of polychlorinated biphenyls and organochlorine pesticides in snow in Eastern Siberia. *Atmosphere* **2022**, *13*, 2117.
43. Bulygina, O.N.; Rasuvaev, V.N.; Trofimenko, L.T.; Schvets, N.V. The Description of the Database of Month Air Temperature at Stations in Russia. Certificate of State Registration of the Database No 2014621485. Available online: http://meteo.ru/data/156-temperature (accessed on 22 August 2023). (In Russian).
44. Bulygina, O.N.; Razyvaev, V.N.; Korshunova, N.N.; Schvets, N.V. The Description of the Database of Month Volumes of Precipitations at Stations in Russia. Certificate of State Registration of the Database No 2015620394. Available online: http://meteo.ru/data/158-total-precipitation (accessed on 22 August 2023). (In Russian).
46. Apasova, E.G.; Kleschenko, L.K. The Description of the Database of Sums Months Sunshine Duration at Stations in Russia. Certificate of State Registration of the Database No 2015621446. Available online: http://meteo.ru/data/160-sunshine-duration (accessed on 22 August 2023). (In Russian).
62. Climatological Standard Normals (1991–2020). Available online: https://www.nodc.noaa.gov/archive/arc0216/0253808/2.2/data/0-data/Region-2-WMO-Normals-9120/ (accessed on 4 October 2023).
63. Climatological Reference Normals (1961–1990). Available online: https://www.ncei.noaa.gov/pub/data/normals/WMO/1961-1990/RA-II/ (accessed on 4 October 2023).
101. Ivanov, V.; Sandell, E. Characterization of polychlorinated biphenyl isomers in Sovol and Trichlorodiphenyl formulations by high-resolution gas chromatography with electron capture detection and high-resolution gas chromatography—Mass spectrometry techniques. *Environ. Sci. Technol*. **1992**, *26*, 2012–2017.

Disclaimer/Publisher's Note: The statements, opinions and data contained in all publications are solely those of the individual author(s) and contributor(s) and not of MDPI and/or the editor(s). MDPI and/or the editor(s) disclaim responsibility for any injury to people or property resulting from any ideas, methods, instructions or products referred to in the content.
